# Supplementary figures and images for: TfR1 facilitates influenza virus endocytosis and uncoating by interacting with NA and M1 via extracellular and intracellular domains
Source: PLoS Pathog. 2025 Oct 10;21(10):e1013511. doi: 10.1371/journal.ppat.1013511 (PMC12533913; doi:10.1371/journal.ppat.1013511)

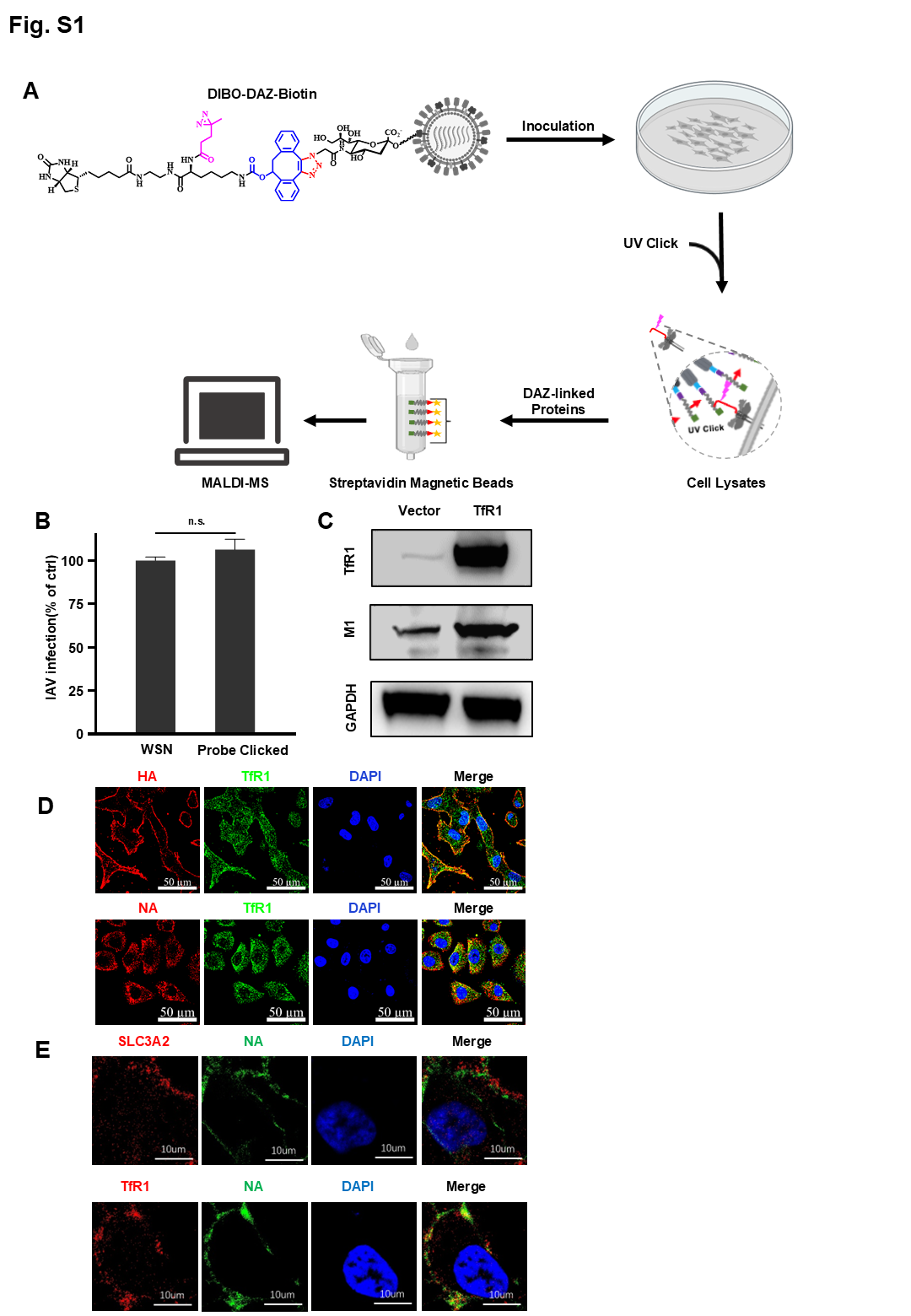

Supplement: S1 Fig — (A) Diagram illustrating the capture of factors interacting with influenza virus using photo-crosslinking and UPLC-MS. Azido-displayed IAV (IAV-N3) was created by converting Ac4ManNAz into terminal sialic acid, and the photo-crosslinking probe was conjugated through a click reaction. TfR1 was captured through UV irradiation, followed by streptavidin enrichment and subsequent identification in proteome analysis. Schematic created using BioRender (https://Biorender.com) (B) Modification of IAV envelope proteins using DIBO-DAZ-Biotin probe did not display any discernible impact on IAV infectivity. A549 cells were infected with probe labelled virus for 12 hours and viral infectivity was confirmed by RT-qPCR. (C) Western blotting characterization of the effect of TfR1 overexpression in Lec1 cells on IAV infection (MOI = 0.5). Equal loading was confirmed by detecting GAPDH. (D) Colocalization analysis of TfR1 with influenza virus envelope proteins HA/NA during IAV endocytosis. A549 cells were infected with IAV (MOI = 0.5) for 2 hours, then fixed and stained with viral antibodies. The Pearson’s Coefficient between TfR1 and HA/NA was 0.928/ 0.802. HA/NA in red, TfR1 in green, nuclei in blue. Scale bars, 50 μm. (E) Colocalization analysis of TfR1/SLC3A2 with influenza virus envelope proteins NA during IAV endocytosis. A549 cells were infected with IAV (MOI = 5) for 2 hours, then fixed and stained with viral antibodies. TfR1/SLC3A2 in red, NA in green, nuclei in blue. Scale bars, 10 μm. Unpaired t-tests were used for statistical analysis, denoting significance as follows: *p < 0.05; **p < 0.01; ***p < 0.001; and ****p < 0.0001, while “n.s.” indicates non-significance. (TIFF) [file ppat.1013511.s001.tiff]

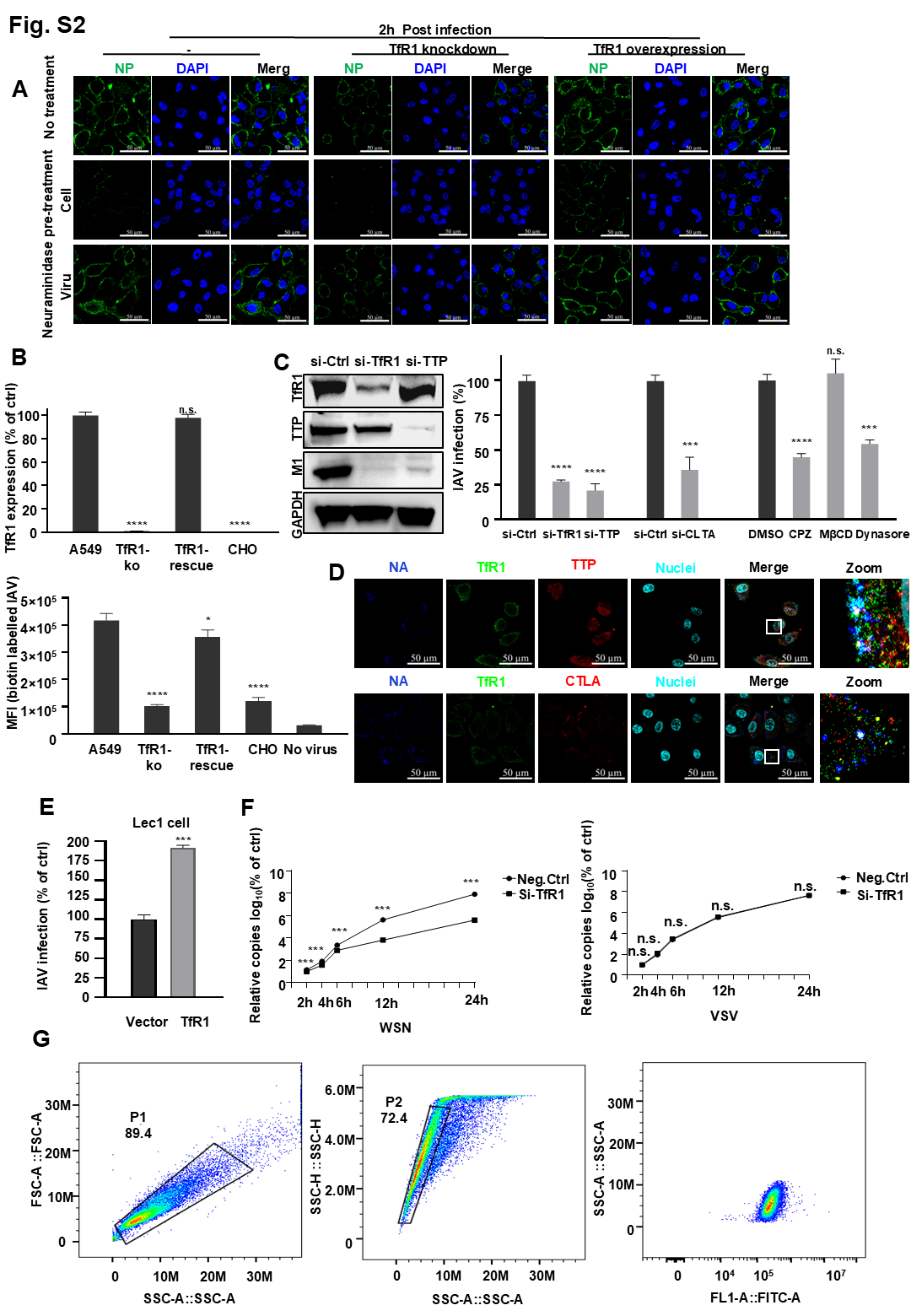

Supplement: S2 Fig — (A) Comparison of sialic acid and TfR1 as potential receptors in mediating IAV entry. A549 cells were transfected with TfR1/vector plasmid or TfR1 siRNA, treated with neuraminidase/BSA at 37°C for 2 hours, and infected by IAVs (MOI = 0.5), which were pre-treated with neuraminidase or untreated. Additionally, influenza virus (MOI = 0.5) was treated with neuraminidase/BSA in glycol buffer at 37°C for 2 hours before adding to A549 cells. Immunofluorescence was used to visualize IAV infection, with NP in green, nuclei in blue. Scale bars, 50 μm. (B) Flow cytometry analysis of surface TfR1 expression and its correlation with the promotion of IAV endocytosis in wild-type, TfR1 knockout, TfR1 rescued A549 cells, and CHO cells. Mean fluorescence intensity was used for further quantification. (C) Assessment of TfR1‘s involvement in initiating the clathrin-mediated pathway (CMP) in IAV endocytosis. A549 cells were either transfected with siRNA targeting TTP or CLTA or treated with various endocytosis inhibitors, followed by infection with IAVs (MOI = 0.5). Subsequent quantification was conducted using western blotting or RT-qPCR. Equal loading was confirmed by detecting GAPDH. Unpaired t-tests were used for statistical analysis, denoting significance as follows: *p < 0.05; **p < 0.01; ***p < 0.001; and ****p < 0.0001, while “n.s.” indicates non-significance. (D) Visual depiction of the further colocalization of NA and TfR1 (seen S1D Fig) with downstream essential factors (TTP/CLTA) in the clathrin-mediated pathway during IAV endocytosis. NA in blue, TfR1 in green, TTP/CLTA in red, Nuclei in cyan. This colocalization, consistent with data in S1D Fig, provides direct visual evidence for the role of TfR1 in mediating IAV endocytosis. Scale bars: 50 μm. Pearson’s coefficients (calculated from zoomed-in regions): NA-TfR1 = 0.672, NA-TTP = 0.689, NA-CLTA = 0.701, TfR1-CLTA = 0.744. (E) Comparative evaluation of sialic acid versus TfR1 as potential receptors in mediating IAV entry. [file ppat.1013511.s002.tiff]

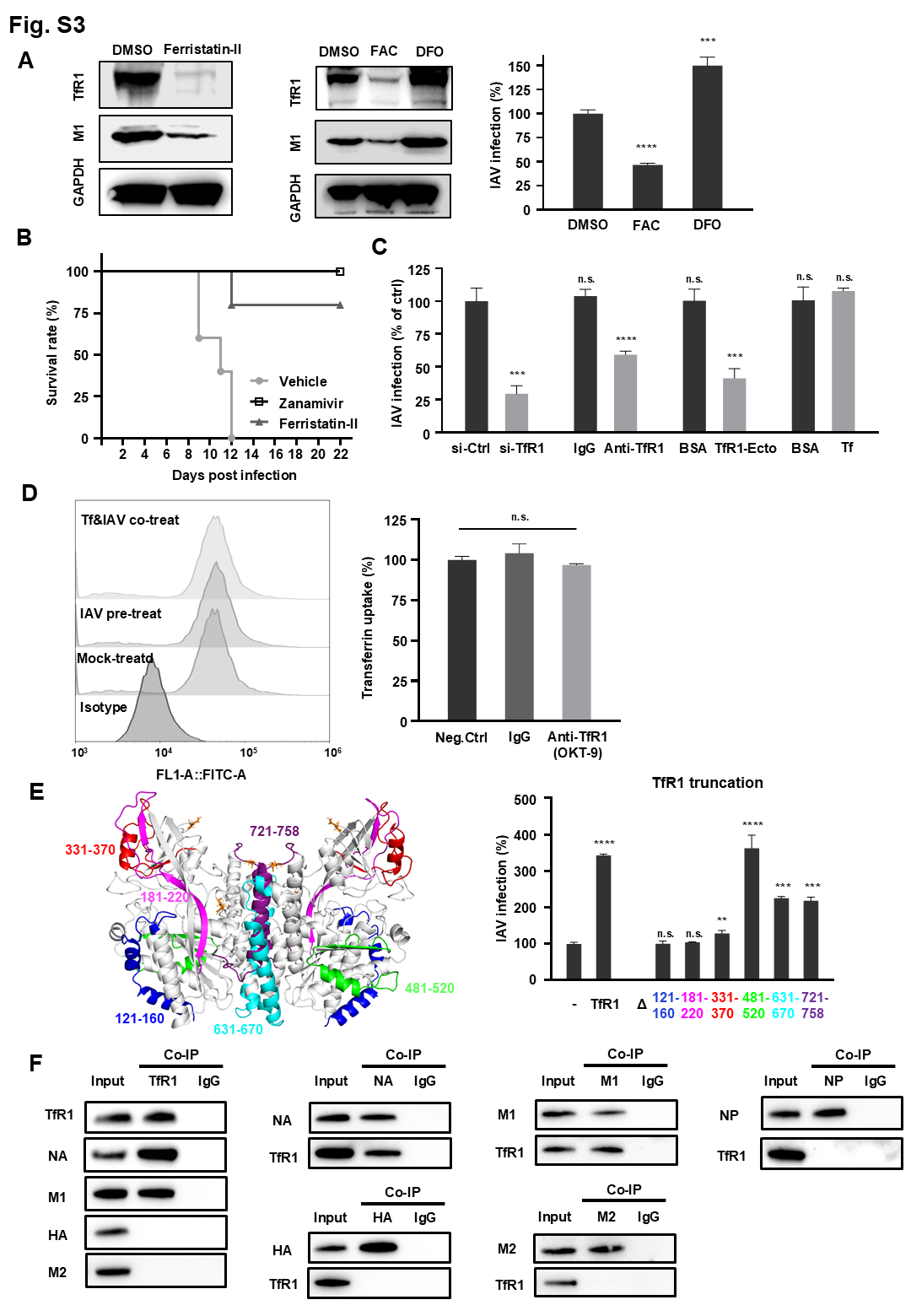

Supplement: S3 Fig — (A) Modulation of TfR1 expression through treatment with various small molecule compounds either restricted or promoted IAV infection. A549 cells were treated with Ferric ammonium citrate/Deferoxamine/Ferristatin-II (100 μM) or an equivalent volume of DMSO for 24 hours, followed by infection with influenza virus (MOI = 0.5). Viral M1 protein was quantified using western blotting or RT-qPCR. Equal loading was confirmed by detecting GAPDH. (B) Evaluation of a small molecule degradator targeting TfR1 in a mouse model. BALB/c mice were divided into three groups (5 mice/group). Mice were intraperitoneally administered Ferristatin-II (20mg/kg) or vehicle (1% DMSO in PBS) daily for 5 days, then challenged with 5x LD50 A/WSN/1933 (H1N1). The survival rate of the mice was monitored and recorded. (C) Disruption of the TfR1-IAV interaction using anti-TfR1 antibody or TfR1-ectodomain protein significantly impaired IAV infection (MOI = 0.5), contrast to TfR1‘s ligand transferrin (targeting the helical domain) (2 mg/mL) for 6 hours. The virus titer was quantified using RT-qPCR. (D) Neither the binding of influenza virus nor the antibody targeting the TfR1 apical domain (OKT-9) interfered with its ligand transferrin uptake. A549 cells were incubated with AF568 labelled transferrin in the presence of OKT-9 or IAV (MOI = 0.5) for 6 hours, transferrin uptake was quantified via flow cytometry. (E) Illustration demonstrating different extracellular truncated TfR1 variants (distinctly colored) and their respective associations with facilitating IAV infection in CHO cells. Various truncated TfR1 plasmids were transfected into CHO cells 48 hours before IAV infection (MOI = 0.5). Virus infectivity was determined by RT-qPCR and compared with vector. (F) Identification of viral components interacting with TfR1 through co-immunoprecipitation assay and western blotting. Lysates from A549 cells at 6 hours post-infection with IAV (MOI = 20) were co-immunoprecipitated using anti-TfR1 antibody alo [file ppat.1013511.s003.tiff]

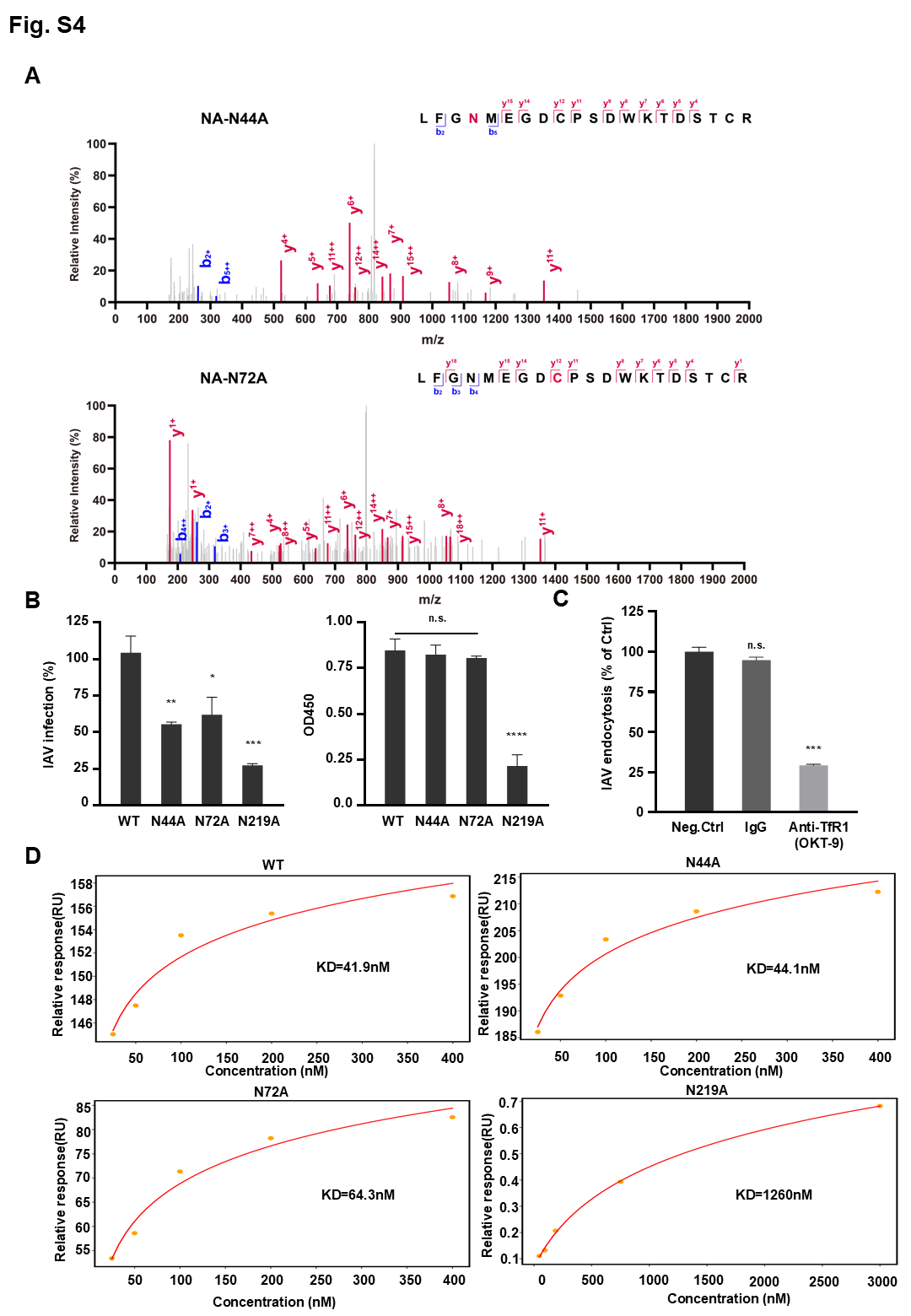

Supplement: S4 Fig — (A) Identification of photo-crosslinked sequences and specific crosslinked sites within TfR1 was achieved using high-resolution mass spectrometry (HRMS). TfR1 protein was incubated with a cleavable photo-crosslinking probe labeled with the mutant IAV at NA-N44A/N72A. Following UV irradiation and SDS-PAGE separation, the sample underwent UPLC-HRMS for further analysis. (B) Characterization of the key glycosylation sites on NA interacting with TfR1 was performed using entry assays and ELISA. TfR1 was immobilized on plates prior to the assays. (C) OKT-9 antibody attenuated IAV infection by specifically binding to the apical domain of TfR1. A549 cells were infected with NHS-SS-biotin labelled IAV (MOI = 0.5) in the presence of OKT-9 or IgG ctrl for 6 hours and then IAV particles uptake was quantified via flow cytometry. (D) Characterization of TfR1’s affinity with IAV NA WT/mutant proteins using Surface Plasmon Resonance (SPR) experiments. NA WT/ mutant proteins were immobilized on CM5 chips and interacted with TfR1 protein to measure the binding affinity constants. Unpaired t-tests were conducted for statistical analysis, and significance levels were indicated as follows: *p < 0.05; **p < 0.01; ***p < 0.001; and ****p < 0.0001, while “n.s.” denoted non-significance. (TIFF) [file ppat.1013511.s004.tiff]

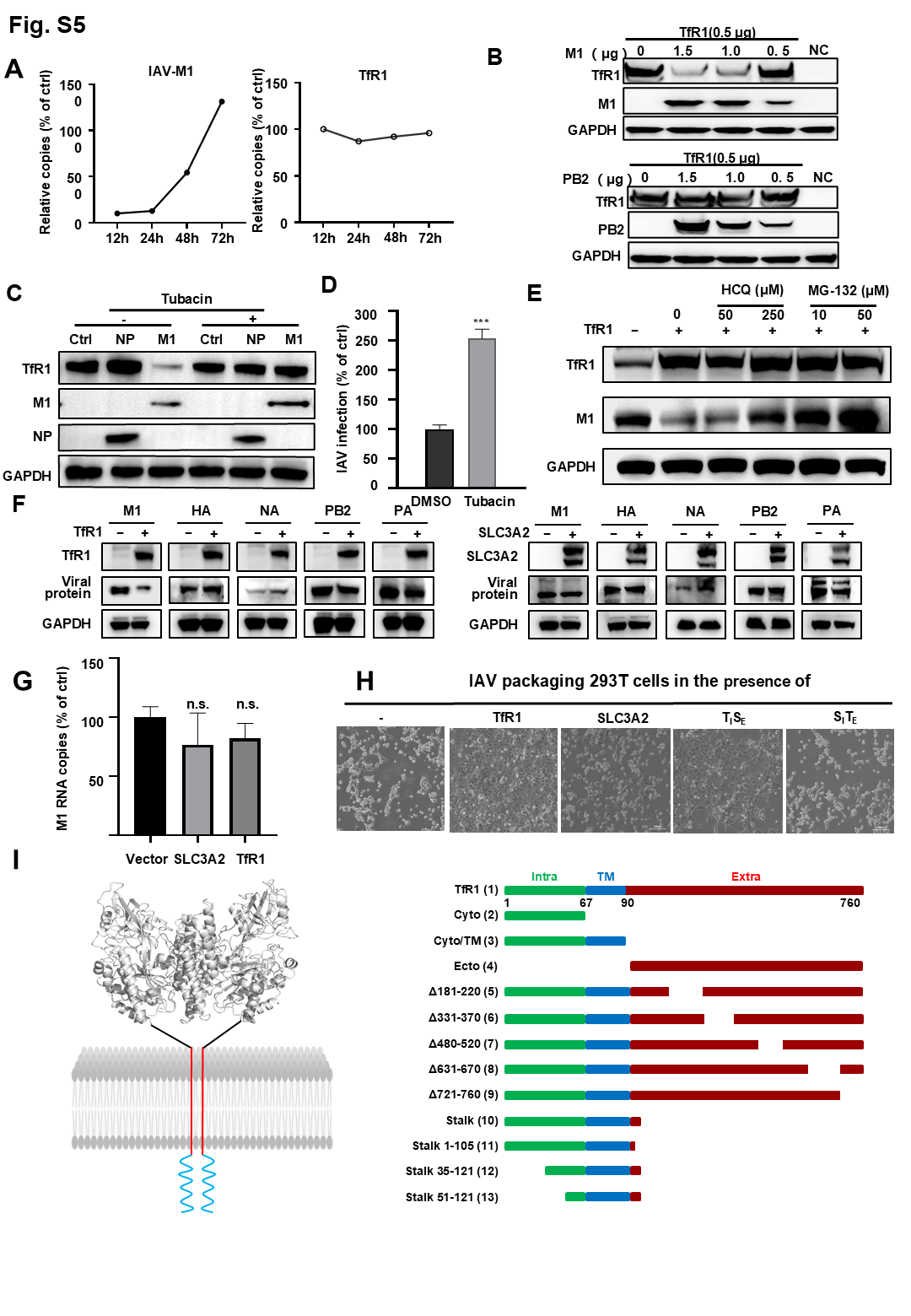

Supplement: S5 Fig — (A) Prolonged exposure to influenza virus (MOI = 0.1) did not exhibit an impact on TfR1 mRNA levels, as assessed through RT-qPCR. (B) Assessment of the impact of viral M1 protein on TfR1 by co-transfecting the TfR1 plasmid (0.5 μg) with varying concentrations of M1 or PB2 plasmid in 293T cells for 48 hours. Equal loading was confirmed by detecting GAPDH. (C) Investigation into the involvement of aggresome systems in TfR1-mediated degradation of the M1 protein. 293T cells were co-transfected with the TfR1 plasmid and viral plasmids expressing M1 or NP in the presence or absence of Tubacin (5 μM). Protein levels of TfR1, M1, and NP were assessed by western blotting 48 hours post-transfection. Equal loading was confirmed by detecting GAPDH. (D) Investigation into the involvement of aggresome systems in virus infection. A549 cells were infected with IAV (MOI = 0.1) in the presence or absence of Tubacin (5 μM) for 12 hours and viral infectivity was confirmed by RT-qPCR. (E) Examination of the interplay between lysosomal and proteasomal systems in TfR1-driven degradation of M1 protein. 293T cells were co-transfected with the M1 expression plasmid and TfR1 expression plasmid, in the presence or absence of HCQ (50 μM, 250 μM) or MG-132 (10 μM, 50 μM). Protein levels of TfR1 and M1 were evaluated by western blotting 48hours post-transfection. Equal loading was confirmed by detecting GAPDH. (F) Western blotting analysis to determine the specificity of TfR1-mediated viral protein degradation. Individual plasmids from the IAV plasmid complex were co-transfected with the TfR1 plasmid for 48 hours, employing the SLC3A2 expression plasmid as a control background. Equal loading was confirmed by detecting GAPDH. (G) RT-qPCR analysis to exclude plasmid competition TfR1-mediated viral protein downregulation. 293T cells were co-transfected with the TfR1/SLC3A2/Vector plasmid for 48 hours. The M1 RNA level was was confirmed by RT-qPCR (H) The critical role of the intracellular domain of [file ppat.1013511.s005.tiff]

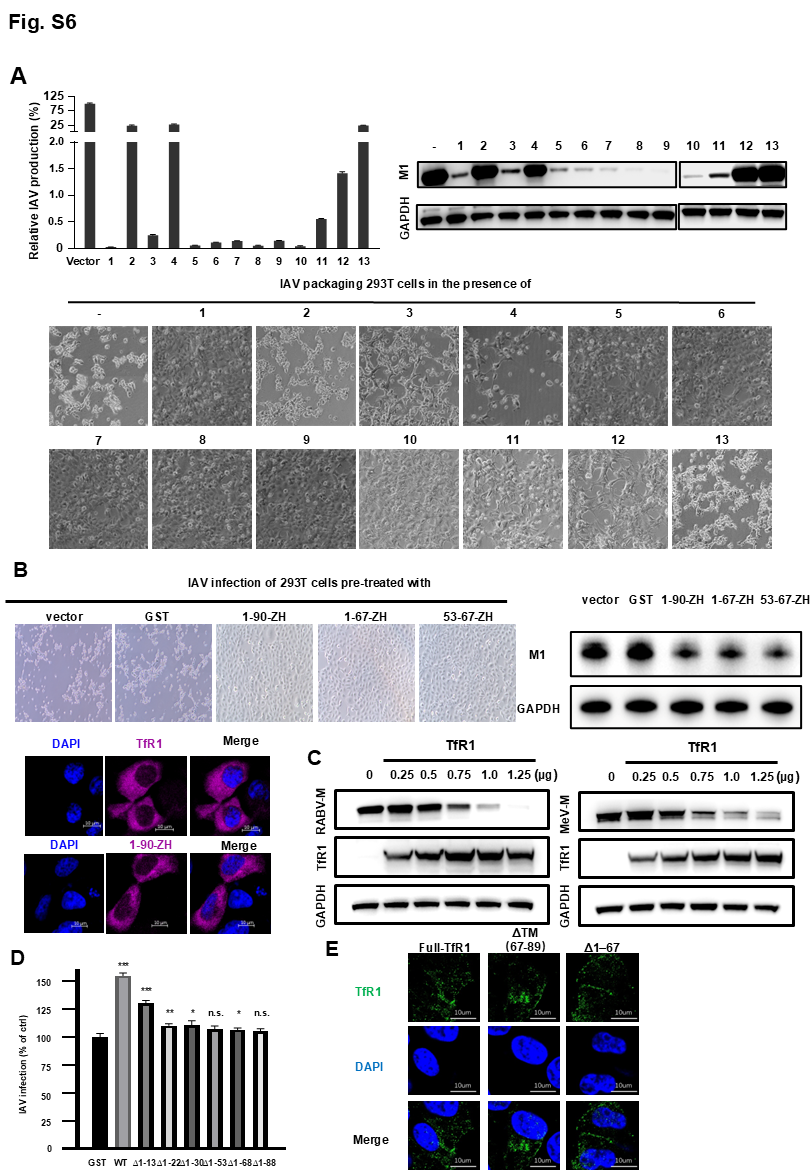

Supplement: S6 Fig — (A) The distinct effects of various TfR1 truncations on IAV packaging were observed. 293T cells were transfected with the 12 IAV package plasmids complex and different TfR1 expression plasmid fragments. Cell state images were captured 3 days after transfection. The supernatant containing IAVs was lysed and subsequently analyzed using RT-qPCR or western blotting. Equal loading was confirmed by detecting GAPDH. (B) Demonstrating the substantial antiviral effect of dimerized truncated TfR1. 293T cells were transfected with expression plasmids for truncated TfR1 containing an isoleucine zipper or GST as control, followed by influenza virus infection (MOI = 0.1). Visual representations of resulting cell cytopathic effects (CPE) were captured in images. Protein levels of M1 were assessed via western blotting 48 hours post-transfection. Equal loading was confirmed by detecting GAPDH. Immunofluorescent staining depicting 293T cells transfected with TfR1 or truncated TfR1, TfR1 or truncated TfR1 (violet), Nuclei (DAPI, blue). (C) Illustrating the dose-dependent degradation of RABV-matrix and MeV-matrix proteins by TfR1. Co-transfection involved RABV-matrix/MeV-matrix expression plasmids with escalating concentrations of TfR1 plasmid in 293T cells, followed by analysis through western blotting 48 hours after transfection. Equal loading was confirmed by detecting GAPDH. (D) Assessment of the effect of TfR1 truncation on virus entry by various intracellularly truncated versions of TfR1. CHO cells were transfected with truncated TfR1 plasmids, followed by influenza virus infection (MOI = 0.5). Infected cells were lysed and quantified by RT-qPCR after 6 hours. (E) Subcellular localization of truncated TfR1 mutants.293T cells were transfected with expression plasmids encoding full-length TfR1 or truncated TfR1 variants (ΔTM and Δ1–67). Cells were fixed and subjected to immunofluorescence staining, TfR1 or truncated TfR1(Green), Nuclei (DAPI, blue). Unpaired t-tests were performed [file ppat.1013511.s006.png]
