# Supplementary material for: TfR1 facilitates influenza virus endocytosis and uncoating by interacting with NA and M1 via extracellular and intracellular domains
Source: PLoS Pathog. 2025 Oct 10;21(10):e1013511. doi: 10.1371/journal.ppat.1013511 (PMC12533913; doi:10.1371/journal.ppat.1013511)
Supplement: Attachment — Submitted filename: PPATHOGENS_D_24_01941_Reviewer Comments_v3.docx [file ppat.1013511.s008.docx]

**Manuscript No.:** PPATHOGENS-D-24-01941

**Title:** TfR1 facilitates influenza virus endocytosis and uncoating by interacting with NA and M1 via extracellular and intracellular domains

**Authors:** X. Wang., Y. Li., X. Wang., K. Guo., Y. Wang., M. Wang., X. Zhu., C. Qin., Y. Mu., Y. Zhang., Z. Chen., T. Yuan., X. Zhang., Q. He., D. Ji., S. Xiao., L. Zhang., D. Zhou

**Summary**

The studies performed and described by the authors aimed to explore non-canonical mechanisms of influenza A virus (IAV) entry and fusion mediated by host transferrin receptor 1 (TfR1). Using UV photo-crosslinking and synthetic TfR1 expression in infected and plasmid-transfected mammalian cells, the authors demonstrated that TfR1 has a greater effect on IAV entry than attachment. Furthermore, TfR1 also has novel functionality in degrading M1 through the proteasome-aggresome signaling pathway, aiding in viral uncoating. Novel aspects of the presented work include the use of metabolic labelling and click chemistry to label IAV particles, and the engineering of TfR1 mutants to assess TfR1 interactions with NA and M1. The conclusions from these studies are scientifically sound and provide more insight into the role of TfR1 in negative-sense RNA virus replication.

**Major Questions/Comments**

- Why use qRT-PCR to assess virus attachment instead of a plaque assay?
- Why were different multiplicity of infection (MOI) values used as opposed to a set standard MOI for all virus infection assays?
- While the data presented in figure 4 panels A-D are convincing, it could be stronger if the biological effects assayed were also evaluated at the transcriptional level using qRT-PCR. There is the possibility that depending on how much plasmid is introduced that the effect shown in Western blot could be due to plasmid competition as opposed to an actual biological effect.
- The introduction of the HDAC6 inhibitor tubacin offers another layer of intricate complexity to role of TfR1 in IAV particle packaging. Have the authors performed experiments seeing whether treating IAV-infected cells with tubacin yields similar results to IAV plasmids transfected into HEK293T cells?
- In figure 6 panel D, infecting cells with IAV instead of using IAV plasmids could provide more support that plasmid competition is not a factor impacting the results of TfR1’s effects on IAV replication.

**Minor Questions/Comments**

- Which statistical analysis method was used to determine that the rescue of TfR1 and M1 in the presence of the proteasome inhibitor MG132 was significant as stated in the text? Statistical analysis to support that statement would strengthen the argument that the proteasome-aggresome pathway plays a role in TfR1-mediated degradation of M1.
- There are inconsistencies in the text when discussing the different IAV strains and metabolically-labeled IAV particles. In the figure 2 legend for panels A and B, it is unclear whether wild-type IAV or metabolically-labelled IAV particles were used. In panel C of the same figure legend, the strains used were clearly stated.
- For figure 2 panels F and G, the flow cytometry gating strategies used to curate and interpret data should be placed in the supplementary section or made available online.
- Figure 2 panel E is confusing. It would be better to have two plots: one plot with the data on A549 cells incubated with neuraminidase and a second plot with the data on IAV incubated with neuraminidase. The data with Lec1 cells could be placed in the supplemental data section.
- The data in figure 3 panel A could be better curated. The smaller Western blot panels showing TfR1 expression with one IAV protein could be placed in the supplemental data section since the larger Western blot panel is descriptive enough.
- The representative model in figure 6 panel F provides a helpful visual that ties in the key findings described in the paper.
