# Supplementary material for: TfR1 facilitates influenza virus endocytosis and uncoating by interacting with NA and M1 via extracellular and intracellular domains
Source: PLoS Pathog. 2025 Oct 10;21(10):e1013511. doi: 10.1371/journal.ppat.1013511 (PMC12533913; doi:10.1371/journal.ppat.1013511)
Supplement: Attachment — Submitted filename: Response to Reviewers.docx [file ppat.1013511.s010.docx]

Dear Editors,

We would like to sincerely thank you and the reviewers for the kind consideration and constructive comments on our manuscript titled " TfR1 facilitates influenza virus endocytosis and uncoating by interacting with NA and M1 via extracellular and intracellular domains" (PPATHOGENS-D-24-01941). We greatly appreciate the constructive feedback, which has helped improve the quality of our work. Below in yellow, we provide point-by-point responses to the reviewers' comments, and have revised the manuscript accordingly with the changes in the revised manuscript been highlighted.

We hope these changes will strengthen our manuscript and the revisions have addressed the concerns raised.

**Editor’s comments:**

*The issues raised regarding overstatements in the manuscript and the need to re-write parts of the manuscript would need to be addressed.*

Thank you for raising this critical point. To avoid overstatements, we have thoroughly revised the manuscript, including re-writing parts of the manuscript, toning down the statement regarding the mechanism description, in particular, using words “support, suggest, unveils, clearly etc.” to replace “indicate, demonstrate, hint, possibly etc.”. More details please refer to the revised manuscript.

*Also, the reviewers raise serious concerns about the data including:*

*- How can Trf1 be essential for infection but also act as an antiviral?;*

We have revised our manuscript to clarify our points. In particular, in the abstract we have emphasized “the reciprocal degradation of TfR1 and M1 not only illuminates a cellular pathway that enriches our understanding of viral entry mechanisms but also presents exciting avenues for the development of innovative antiviral strategies” (1^st^ page); in the main text we have revised as “Given the IAV matrix protein as an important component for progeny virus production, TfR1-mediated degradation of M1 provides an exciting avenue for the development of novel antivirals. We first evaluated the broad-spectrum effect of TfR1-mediated degradation on the viral matrix protein, spanning antigenically distinct influenza strains. We found significant degradation of matrix proteins by TfR1 extended to A/Puerto Rico/8/1934 (H1N1), A/Aichi/2/1968 (H3N2), and A/Anhui/1/2013 (H7N9), attributed to 92% sequence homology in M1 among these distantly related influenza strains (**Figure 6C**). The influence of TfR1-mediated M1 degradation also displayed a clear dose-dependent inhibition of IAV progeny virus production from both IAV plasmids complex-transfected cells and infected cells with IAV (**Figure 6D**).” (page 10)

Additionally, we provide a representative model in **Figure 6F** that provides a helpful visual that ties in the key findings - one stone two birds function of TfR1 exploited by influenza virus for IAV entry - described in the paper.

*- The authors used a late time point of infection rather than an early time point to observe viral entry and hence the experiments should be repeated at an earlier time point;*

Based on editor’s and reviewer’s comments, we have repeated the co-immunoprecipitation experiments after 2 and 6 hours, respectively, post-infection at a MOI of 20. We found that NA and M1 were still co-immunoprecipitated by TfR1 to a little different extent; this suggests that within 24 hours the interaction of TfR1 with NA and M1 still exists. We revised the manuscript accordingly; more details please refer to the revised manuscript (page 6).

*- There are many control experiments missing including on viral entry and on the use of treatments such as Tubacin.*

Based on editor’s and reviewer’s comments, the control experiment (NaN_3_ as an endocytosis inhibitor) in viral entry assay has been added. The function regarding Tubacin usage has been discussed in the revised manuscript. Other missing controls such as protein markers and Western blotting loading control (GAPDH) have also been added. More details please refer to the revised manuscript (page 5).

*- The experiments should be repeated with different IAV strains,*

Based on editor’s comments, the experiments regarding TfR1 as a potential entry receptor for influenza virus and as a potential antiviral drug have been repeated with different IAV strains, including A/WSN/1933 H1N1, A/PR/8/1934 H1N1, and A/Aichi/2/1968 H3N2. More details please refer to the revised manuscript (page 4; page 10).

*- The quality of figures and explanations needs to be improved.*

Based on editor’s and reviewer’s comments, partial figures, figure legends and explanations have been revised to clarify main points of the manuscript. More details please refer to the revised manuscript.

**Reviewer #1:**

**Major Issues：**

*1.	Figure 2F: A control experiment is missing to show that the assay can discriminate between attached and internalized virus. After 2 hours at 37°C, some virus will remain attached to the cell surface. The authors should include controls such as endocytosis inhibitors or biotinylated virus with and without permeabilization to ensure that they are measuring only internalized virus (see PMID: 26575457 for relevant methods)*

Thank you for raising this critical point. To ensure that only internalized virus is measured, we have re-designed this experiment, based on the methods recommended by the reviewer (PMID: 26575457), by adding endocytosis inhibitor NaN_3_ as a control. We found very less virus (5~10%) was detected even after culturing at 37°C for 2 hours, suggesting the endocytosis of attached viruses was also blocked. Additionally, we have further investigated viral internalization by using A549 cells, TfR1-knockout A549 cells, and CHO cells which were exposed to sulfo-NHS-SS-Biotin-labeled IAVs under identical conditions. Flow cytometry analysis showed an ~80% reduction in mean fluorescence intensity in TfR1-knockout cells compared to parental A549 cells.

The updated manuscript is presented as follows: “To evaluate TfR1's role in the endocytosis of attached virus particles, we transferred the aforementioned cells from 4°C to 37°C and incubated them for an additional two hours with or without NaN_3_, a viral endocytosis inhibitor. RT-qPCR analysis revealed that TfR1-knockdown cells exhibited a ~70% reduction in the number of internalized viruses compared to control cells (**Figure 2F** and **Figure S2G**). The viral internalization was further investigated by exposure of A549 cells, TfR1-knockout A549 cells, and CHO cells to sulfo-NHS-SS-Biotin-labeled IAVs [37] (MOI=5) under identical conditions. Flow cytometry analysis, using AF488-conjugated streptavidin [36], showed an ~80% reduction in mean fluorescence intensity in TfR1-knockout cells compared to parental A549 cells (**Figure S2B**). This reduction was comparable to that observed in CHO cells, which inherently lack TfR1 expression. Moreover, overexpression of TfR1 in TfR1-knockout A549 cells restored IAV endocytosis (**Figure 2G**, **Figure S2B** and **Figure S2G**). Thus, it seems likely that TfR1 is more crucial for IAV endocytosis than for initial attachment to the cell surface, despite they are not independent steps.” (page 5)

*2.	Figure 3A: The co-immunoprecipitations are performed after 24 hours of infection, but it would be more informative to perform these experiments shortly after cold binding or early post-infection (e.g., 1-2 hours post-infection) at a high MOI (e.g. MOI=10 or 20) to test for interactions. This applies to the entire manuscript.*

Thank you for raising this critical point. Accordingly, we have repeated the co-immunoprecipitation experiments after 2 and 6 hours, respectively, post-infection at a MOI of 20. We found that NA and M1 were still co-immunoprecipitated by TfR1 to a little different extent; this suggests that within 24 hours the interaction of TfR1 with NA and M1 still exists.

The updated manuscript is presented as follows: “Subsequently, co-immunoprecipitation experiments were conducted using lysates from infected A549 cells to identify viral components interacting with TfR1. We found viral NA and M1 proteins were selectively co-immunoprecipitated with the anti-TfR1 antibody at either 2, 6 or even 24 hours post-infection, whereas HA and M2 proteins didn’t exhibit such interaction (**Figure 3A** and **Figure S3F**). These interactions between TfR1 and NA, as well as M1, were further confirmed through co-immunoprecipitation using anti-NA and anti-M1 antibodies. In contrast, neither anti-HA nor anti-M2 antibodies co-immunoprecipitated with TfR1.” (page 6)

*3. Figure S4B: The loss of binding to TfR1 by the NA mutant N219A should be confirmed using surface plasmon resonance (SPR). In addition, virus entry studies should be performed with a recombinant virus harboring the N219A mutation in NA, to confirm its role in TfR1-mediated entry and uncoating.*

Thank you for raising these critical points. In fact, we have previously used surface plasmon resonance to validate the loss of binding of the NA mutant N219A virus to TfR1. Unfortunately, not always consistent and convincing data were obtained but significant variations. Instead, we have performed SPR experiments using NA mutant N219A protein rather than virus with TfR1 immobilized on chip. A comparison of KD values of TfR1 to WT NA vs. to N219A disclosed a noticeable increase, supporting the binding of TfR1 to NA via the N219 residue.

We also assessed NA mutant virus in viral entry, only the N219A variant displayed a discernible reduction in viral entry (**Figure S4B**), highlighting the crucial role of NA glycosylated N219 residue in its interaction with TfR1.

The updated manuscript is presented as follows: “Concerning the potential glycosylation site of NA (N44, N72, N219) for the decorating probe, site-specific mutation and infectivity experiments showed that only the N219A variant displayed a discernible reduction in viral infection (**Figure S4B**), highlighting the crucial role of NA glycosylated N219 residue in its interaction with TfR1. Consistently, the crosslinked peptide spanning from 345 to 364 was detected in NA mutations N44A and N72A but absent in IAV featuring the N219A mutation (**Figure 3E and Figure S4A**). ELISA results also confirmed this observation, demonstrating a noticeable decrease in binding affinity between the N219A variant and TfR1, which has been immobilized (**Figure S4B**). Furthermore the OKT-9 antibody attenuated IAV endocytosis by specifically binding to the apical domain of TfR1 (Figure S4C). A comparison of the KD value of TfR1 to WT NA vs. to N219A disclosed a substantial increase trend, supporting the binding of TfR1 to NA potentially via the N219 residue (**Figure S4D)**.” (page 7)

*4. Figure 5B: Expression controls for the chimeric constructs are missing and should be included.*

Thank you for this valuable comment. Accordingly, anti-FLAG-tag as an expression control has been added in Western blotting analysis in Figure 5B.

The updated manuscript is presented as follows: “Co-transfection of the M1 plasmid with TISE still resulted in M1 degradation, while repeating the co-transfection with SITE led to no M1 degradation (**Figure 5B** and **Figure S5H**), indicating the intracellular domain of TfR1 as the trigger for M1 degradation. A FLAG-tag has been engineered into the chimeric constructs as an expression control.” (page 9)

*5. Figure 5C: The effect of TfR1 truncation on virus entry should be investigated and discussed.*

Thank you for raising this critical point. Accordingly, we have designed experiments to evaluate the effect of TfR1 truncation, mainly at the cytosolic domain including ∆1-13, ∆1-22, ∆1-30, ∆1-53, ∆1-67, ∆1-88, on virus entry. We found that transfection of most TfR1 variants to CHO cells, which naturally lack of TfR1, failed to restore their susceptibility to IAV infection, likely due to the loss of the endocytic signal in the intracellular domain. Clearly, it is the extracellular domain of TfR1 that functions as a receptor and initiates viral endocytosis.

The updated manuscript is presented as follows: “Successive removal of the N-terminal residues still led to a significant M1 reduction and this effect persisted until deletion of residue 53; further deletions beyond residue 54, i.e. the transfer-stop sequence adjacent to the transmembrane domain, diminished its capability to degrade M1 and removal of residues 1-63 entirely eliminated its degradation capability (**Figure 5D**). In a parallel experiment evaluating the effect of TfR1 truncation on viral entry, we found that none of the truncated variants restored the susceptibility of CHO cells to IAV infection (**Figure S6D**). Clearly, it is the intracellular transfer-stop sequence of TfR1 together with the that triggers viral matrix M1 degradation.” (page 10)

*6. Figure 6D: Conflicting and confusing results with data presented in previous Figures where TfR1 promotes IAV infection, here TfR1 inhibits IAV replication in a dose-dependent manner. Virus growth curves on wild type, TfR1 KO and TfR1 over expressing A549 or 293T cells should be performed.*

We are sorry for those vague presentations that make our points confusing. In **Figure 6D** the data represent a quantitative assessment of virus packaging yield in TfR1-transfected cells. A549 cells were co-transfected with 12 IAV packaging plasmids and varying concentrations of TfR1 plasmids, rather than an IAV infection model. The intracellular domain of TfR1 can degrade M1, reducing viral packaging. The difference between the two models explains the observed discrepancy. Additionally, we have included virus growth curves for IAV infection in wild type and TfR1 knock-down A549 cells (**Figure S2F**).

Given the TfR1-mediated degradation of M1, which is the essential component for progeny viruses, we are wondering whether TfR1 can be developed as a novel antiviral targeting M1 to inhibit progeny virus. To this end, we first evaluated the broad-spectrum effect of TfR1-mediated degradation on the viral matrix protein spanning antigenically distinct influenza strains. We then evaluated the dose-dependent TfR1 on inhibition of IAV progeny virus production from both IAV plasmids complex-transfected cells and infected cells with IAV. Infecting cells with IAV instead of using IAV plasmids might exclude transfection and plasmid competition as factors impacting the results of TfR1’s effects on IAV replication. Furthermore, investigations revealed that TfR1-mediated degradation extended beyond influenza viruses, as co-transfection with TfR1 led to degradation of matrix proteins of measles virus (MeV) and rabies virus (RABV) in a dose-dependent manner.

The updated manuscript is presented as follows: “Given the IAV matrix protein as an important component for progeny virus production, TfR1-mediated degradation of M1 provides an exciting avenue for the development of novel antivirals. We first evaluated the broad-spectrum effect of TfR1-mediated degradation on the viral matrix protein, spanning antigenically distinct influenza strains. We found significant degradation of matrix proteins by TfR1 extended to A/Puerto Rico/8/1934 (H1N1), A/Aichi/2/1968 (H3N2), and A/Anhui/1/2013 (H7N9), attributed to 92% sequence homology in M1 among these distantly related influenza strains (**Figure 6C**). The influence of TfR1-mediated M1 degradation also displayed a clear dose-dependent inhibition of IAV progeny virus production from IAV plasmids complex-transfected cells and infected cells with IAV (**Figure 6D**). Furthermore, investigations revealed that TfR1-mediated degradation extended beyond influenza viruses, as co-transfection with TfR1 led to degradation of matrix proteins of measles virus (MeV) and rabies virus (RABV) in a dose-dependent manner (**Figure 6C** and **Figure 6E**). Hence, the discovery of viral matrix protein degradation by TfR1 holds promise for the development of degradative antiviral agents that transcend the scope of influenza viruses.” (page 10)

*7. Figure S6: Localization of TfR1 truncation mutants need to be assessed by immunofluorescence or flow cytometry. Negative controls with expression plasmids (e.g. GST) need to be added to show specificity of the observed effects on M degradation.*

Thank you for raising this critical point. Accordingly, we have designed experiments to localize TfR1 truncation mutants by immunofluorescence. Additionally, a negative control, a GST-expressing plasmid, has been included here to ensure the specificity on the observed M degradation.

The updated manuscript is presented as follows: “We also tried to shorten TfR1 by substituting its extracellular domain and transmembrane sequence with an isoleucine zipper, and the intracellular domain was truncated into various versions (1-90-ZH, 1-63-ZH, and 53-67-ZH). It was found that all of them predominantly existed as dimers and trimers (**Figure 6A**) and exhibited notable efficacy in degrading M1 protein in co-transfection and IAV infection (**Figure 6B** and **Figure S6B**), whereas the GST negative control showed no detectable impact on M1 degradation. Immunofluorescence analysis further confirmed the cytoplasmic localization of these truncation mutants similar to the full-length TfR1 (**Figure S6B**). This suggests that the role of the transmembrane domain is to maintain TfR1 as a dimer to trigger M1 degradation and can be replaced by an isoleucine zipper.” (page 10)

And legend: “(B) Demonstrating the substantial antiviral effect of dimerized truncated TfR1. 293T cells were transfected with expression plasmids for truncated TfR1 containing an isoleucine zipper or GST as control, followed by influenza virus infection (MOI=0.1). Visual representations of resulting cell cytopathic effects (CPE) were captured in images. Protein levels of M1 were assessed via western blotting 48 hours post-transfection. Equal loading was confirmed by detecting GAPDH. Immunofluorescent staining depicting 293T cells transfected with TfR1 or truncated TfR1, TfR1 or truncated TfR1 (violet), Nuclei (DAPI, blue).”

**Minor Issues**

*1.	Figure 1A: The schematic showing IAV plasmids is confusing and does not add clarity to the overall understanding. Replacing this with an unlabeled illustration of an IAV particle would be more helpful.*

We apologize for the unclear labeling of the plasmids in the diagram. The viruses containing Ac4ManNAz was obtained by the virus packaging process (during plasmid transfection, the Ac4ManNAz was added simultaneously), but this step was not clearly conveyed in the original diagram. In the updated diagram, we have adjusted the order of the plasmids and Ac4ManNAz to be presented together and added a schematic of the cells to more clearly explain the process of virus acquisition.

*2.	Figure 1B: The multiplicity of infection (MOI) appears to be inaccurate throughout the manuscript. It appears to be closer to an MOI of 50, or it may indicate the presence of defective nuisance particles. The authors should perform a plaque assay comparing the tagged virus with the wild-type virus to determine if infectivity is affected by the tagging*

Many thanks for carefully revising this manuscript. The MOI here is indeed 50 but not 0.5; the legend has been corrected accordingly. To determine whether the tagged virus has the same infectivity as the wild-type virus, both RT-qPCR and Western blotting approaches instead of the plaque assay have been utilized (**Figure S1B**); the plaque assay does not work consistently in our study.

*3.	Figure 1C: The phrase “WSN package” is unclear. It would be clearer to simply state “WSN”. Text Clarity: The text states that WSN are “co-cultured” with A549 cells. This is incorrect and should be changed to “A549 cells were infected with WSN”.*

Many thanks for your kindness to edit the legend. The edited phrase and text are really what we want. **Figure 1D** legend has thus been revised as “Analysis of the photo-crosslinked products obtained from A549 cells, which were sequentially infected with WSN particles (MOI=50) pre-labelled by DIBO-DAZ-Biotin or untreated for 1h on ice and then transferred to 37°C for 5min.”; “A549 cells were then infected with IAV-DAZ-Biotin WSN particles (MOI=50) at 4°C for 2 hours to facilitate virus attachment, followed by a shift to 37°C for 5 minutes.”

*4.	Figure 1 Panels: The order of panels D and E should be reversed for a more logical flow.*

Based on the reviewer’s suggestion, the order of panels D and E have been reversed accordingly.

*5.	Figure 1D: The molecular weight ladder label is missing, and this oversight occurs in the Western blot (WB) images in the remaining figures. Please ensure that all WBs have properly labeled ladders for reference.*

Many thanks for carefully revising the figures. Accordingly, the molecular weight markers have been added to the Western blot (WB) images.

*6.	Figure 2B: The time post infection should be clearly indicated in this figure to provide context for the data presented.*

Thank you for this valuable comment. Accordingly, the time post-infection has now been indicated in the figure legend.

*7.	Figure 2C: A virus growth curve should be included to complement the data. In addition, a negative control (e.g., vesicular stomatitis virus, VSV) should be included to demonstrate that the effect of TfR1 is specific to IAV.*

Thank you for this valuable comment. A time-course of WSN virus growth curve with VSV as a negative control has been added in the revised manuscript as shown in **Figure S2F**.

The updated manuscript is presented as follows: “This observation was further supported by assessing virus infections of A549 cells with various IAV strains (A/WSN/1933 H1N1, A/PR/8/1934 H1N1, and A/Aichi/2/1968 H3N2), underscoring the critical role of TfR1 across diverse IAV strains (**Figure 2C** and **Figure S2F**).” (page 4)

*8.	Figure 2E: The graph labels are confusing, and the conditions should be clarified to make the data easier to interpret.*

Thank you for raising this critical point. Accordingly, we have revised **Figure 2E** into two plots: one plot with the data on A549 cells incubated with neuraminidase and a second plot with the data on IAV incubated with neuraminidase. The data with Lec1 cells have been placed in the **Figure S2E**.

*9.	Figure 2G: The differentiation between A549 and CHO cells could be improved by using different colors or histogram styles to better illustrate the data.*

Thank you for this valuable comment. Accordingly, different colors have been utilized to better illustrate the data, as shown in the revised **Figure 2G**.

*10.	Figure 2F: Lectin staining could also be used to show that TfR1 does not affect sialic acid levels.*

Thank you for this valuable comment. Unfortunately, the quantified antibody for lectin staining is not available for assessing the sialic acid level on TfR1-knockdown and overexpressed cells, thus we still used RT-qPCR and flow cytometry to quantify the attachment and endocytosis profile. More details please refer to the responses to Major revision 1 above.

*11.	The abbreviation "CLTA" is not introduced in the manuscript and should be explained when first mentioned.*

Thank you for raising this critical point. Accordingly, we added the full name of CLTA (Clathrin light chain A) the first time it mentioned in the manuscript (section 2.2, page 9, **Figure S2D**).

*12.	Figure 3A: Analyzed time points and MOI are not chosen appropriately to study IAV entry.*

Thank you for raising this critical point. We have added co-immunoprecipitation data at 2 and 6 hours, respectively, post-infection using a high MOI (MOI= 20). More details please refer to the responses to Major revision 2 above.

*13.	Figure 3B: The manuscript does not specify how the recombinant proteins (HA, NA, M1, M2, and NP) used for the binding studies were produced or purchased. This information should be clearly provided.*

Thank you for raising this critical point. Accordingly, the source of viral proteins purchased and used in this study has been added in the revised manuscript. (page 29)

*14.	Figure 3C and 3D: The methodological explanations are insufficient to understand how the two TfR1 domains were selected as potential interacting partners with NA. This needs to be clarified.*

Thank you for raising this critical point. The methodological explanations regarding how the two TfR1 domains were selected as potential interacting partners with NA have been revised as follows: “Given TfR1 as one crosslinked protein upon radiation of IAV-DAZ-Biotin WSN particles with A549 cells, we then conducted a parallel photo-crosslinking experiment with TfR1 replacing A549 cells to identify which domains and even residues of TfR1 were engaged with WSN particles. Analysis of photo-crosslinking samples derived from the IAV & TfR1 complex, compared to the non-photo-crosslinking controls, using Q Exactive™ HF-X MS (Thermo) revealed a loss in peptides within the apical domain, spanning residues 209-231 and 340-364 (**Figure 3C**). This suggests that the apical domain of TfR1 may have been covalently crosslinked with WSN virus (NA). In order to identify photo-crosslinking sites, probe incorporating a cleavable diazirine moiety (**Figure 3D**) was utilized, which was designed to undergo acid-induced cleavage (10% formic acid), generating a mass tag (75.05750 Da) on the crosslinked proteins. High-resolution mass spectrometry (HRMS) revealed a precise 72.05750 Da increase in molecular weight, matching the crosslinked diazirine moiety, pinpointed to the peptide sequence 345-LFGNMEGDCPSDWKTDSTCR-364 (calc. [M+H]+=2448.02695 Da, found [M+H]+=2448.04502 Da, Δmass=0.01807 Da and Δppm=7.38, **Figure 3E**). Using this refined approach, we precisely mapped the TfR1-NA interaction to residues 345-364 within the apical domain (sequence: LFGNMEGDCPSDWKTDSTCR). Critically, the diagnostic mass tag localized to G347 and N348 (calc. [M+H]+=2448.02695 Da, found [M+H]+=2448.04502 Da, Δmass=0.01807 Da and Δppm=7.38), confirming these residues as direct crosslinking sites.” (page 7)

*15.	Figure S3D: The term "negative control" is vague. It would be clearer to use "untreated" or "mock-treated" to describe the control group.*

Thank you for this valuable comment. Accordingly, the term "negative control" has been replaced by "mock-treated" to describe the control group.

*16.	Figure S3E: The comparison for the statistical test is unclear. The authors need to specify which columns are being compared.*

Thank you for raising this critical point. More details regarding statistical to the figure legend has been added in the revised **Figure S3E** as follow: (E) Illustration demonstrating different extracellular truncated TfR1 variants (distinctly colored) and their respective associations with facilitating IAV infection in CHO cells. Various truncated TfR1 plasmids were transfected into CHO cells 48 hours before IAV infection (MOI=0.5). Virus infectivity was determined by RT-qPCR and compared with vector. Unpaired t-tests were conducted for statistical analysis, and significance levels were indicated as follows: *p < 0.05; **p < 0.01; ***p < 0.001; and ****p < 0.0001, while "n.s." denoted non-significance. We also made revisions to the labels in the diagram.

*17.	Figure S4B: Details on how the ELISAs were performed with the NA mutant viruses are missing. The authors should explain how the assays were normalized and confirm whether the viruses are viable. In addition, the Y-axis is labelled "% IAV infection," but it is unclear how the ELISA results were translated into infection rates.*

Thank you for raising these critical points and we are sorry for those vague presentations that make our points confusing. Given three glycosylation sites (N44, N72, N219) on NA as the potential site for decorating probe and crosslinking with TfR1, we conducted site-specific mutation experiments to explore which glycosylation sites of NA are engaged. The NA mutant WSN virus at the potential glycosylation site N44A, N72A and N219A were generated and then characterized by virus infectivity and affinity ELISA assay. We found that only the N219A variant displayed a discernible reduction in viral infection (**Figure S4B**), highlighting the crucial role of NA glycosylated N219 residue in its interaction with TfR1. Consistently, the ELISAs assay disclosed a noticeable decrease in binding affinity between the N219A variant and TfR1, which has been immobilized (**Figure S4B**).

The updated manuscript is presented as follows: “Concerning the potential glycosylation site of NA (N44, N72, N219) for the decorating probe, site-specific mutation and infectivity experiments showed that only the N219A variant displayed a discernible reduction in viral infection (**Figure S4B**), highlighting the crucial role of NA glycosylated N219 residue in its interaction with TfR1. Consistently, the crosslinked peptide spanning from 345 to 364 was detected in NA mutations N44A and N72A but absent in IAV featuring the N219A mutation (**Figure 3E** and **Figure S4A**). ELISA results also confirmed this observation, demonstrating a noticeable decrease in binding affinity between the N219A variant and TfR1, which has been immobilized (**Figure S4B**).”

The updated figure legend S4B is presented as follows: “Characterization of the key glycosylation sites on NA interacting with TfR1 was performed using entry assays and ELISA. TfR1 was immobilized on plates prior to the assays.”

*18.	Figure 4B: The experimental design is unclear. Increasing amounts of M1 and a negative IAV protein should be titrated with constant amounts of TfR1 to assess the interaction.*

Thank you for raising these critical points. The experiment involving **Figure 4B** did indeed lack a comprehensive design during the initial manuscript. In the original manuscript, we only fixed the amount of M1 plasmid transfection and gradually increased the TfR1 plasmid amount to detect the dose-dependent mutual degradation of TFR1 and M1. In the new version, we fixed the amount of TfR1 plasmid transfection and gradually increased the M1 plasmid amount, and obtained the same dose-dependent mutual degradation results (**Figure S5B**). This new control experiment has made the results accurate.

The updated manuscript is presented as follows: “Subsequently, in the experiments where we either fixed the amount of TfR1 plasmid transfection and gradually increased the co-transfected M1/PB2 plasmid amount, or fixed the M1/PB2 plasmid transfection amount and gradually increased the co-transfected TfR1 plasmid amount, we demonstrated that elevating TfR1 expression in host cells led to a dose-dependent decrease in M1, but not PB2, protein levels (**Figure 4B** and **Figure S5B**).” (page 8)

*19.	Figure 4G: The authors should include staining for both M1 and TfR1 in this figure.*

Thank you for this valuable comment. Accordingly, staining for both M1 and TfR1 has been added in the revised **Figure 4G**.

*20.	Figure 4H: This figure needs a more detailed explanation of what was transfected and what was measured. The interaction between M1 and wild-type TfR1 is also missing and should be included.*

Thank you for raising this critical point. In fact, this is an in vitro experiment wherein protein M1 was purchased from SinoBiological and proteins TfR1 and its truncated variants were expressed in house. Protein co-aggregation was analyzed with the Abcam Protein Aggregation Assay Kit (ab234048) following the manufacturer's protocol. Aggregation was determined based on the ΔRFU using a standard curve. We initially attempted the Abcam Protein Aggregation Assay Kit (ab234048), but it was discontinued during our study. Unfortunately, the alternative ENZO kit introduced significant technical limitations: Wild-type (WT) TfR1 contains residual detergent (from the purification process), which generated unacceptably high false-positive aggregation signals. Despite extensive optimization, reliable WT TfR1 data could not be obtained. We sincerely apologize for the omission of WT TfR1-M1 interaction data.

*21.	Figure 5B: The choice of domain swap between SLC3A2 and TfR1 is questionable as it may not be the most relevant comparison. It would make more sense to swap domains with a related protein that does not affect M1 degradation. The authors might consider testing transferrin receptors from other species instead.*

Thank you for raising this critical point. In fact, the purpose of this study is to identify the crucial region of TfR1 responsible for initiating M1 degradation. We thus conducted experiments involving the exchange of its extracellular and intracellular domains with a host membrane protein showing no discernible impact on M1 degradation, such as SLC3A2. SLC3A2 was also found in volcano plot analysis after photo-crosslinking. We found that co-transfection of the M1 plasmid with TfR1intracellular-SLC3A2extracellular plasmid still resulted in M1 degradation, while repeating the co-transfection with TfR1extracellular-SLC3A2intracellular plasmid led to no M1 degradation. This indicates that it is the intracellular domain of TfR1 as the trigger for M1 degradation. Consistently, TfR1 truncations showed that truncating any fragment within the extracellular domain had no impact on M1 degradation, while removal of the transmembrane domain, either in conjunction with the extracellular or intracellular domain, abolished M1 degradation.

The updated manuscript is presented as follows: “To identify whether the extracellular or intracellular domain of TFR1 is responsible for M1 degradation, we utilized SLC3A2, a membrane receptor that does not induce M1 degradation (**Figure S5F**). Meanwhile co-transfection of the M1 plasmid with TfR1 or SLC3A2 plasmid had no influence on M1 transcriptional level (**Figure S5G**). We created SITE by fusing the extracellular domain of TFR1 with the intracellular domain of SLC3A2 and TISE by fusing the intracellular domain of TFR1 with the extracellular domain of SLC3A2. . Co-transfection of the M1 plasmid with TISE still resulted in M1 degradation, while repeating the co-transfection with SITE led to no M1 degradation (**Figure 5B** and **Figure S5H**), indicating the intracellular domain of TfR1 as the trigger for M1 degradation.” (page 9)

*22.	Figure 6: This figure is not convincing as presented and requires significant revision or additional data to support the claims.*

Thank you for raising this critical point and we are sorry for those vague presentations that make our points confusing. This figure is regarding the translational potentials of TfR1-mediated M1 degradation. To this end, we first evaluated the broad-spectrum effect of TfR1-mediated degradation on the viral matrix protein spanning antigenically distinct influenza strains, and then evaluated the dose-dependent TfR1 on inhibition of IAV progeny virus production from both TfR1-transfected cells and infected cells with IAV. Furthermore, TfR1-mediated viral matrix protein degradation extended beyond influenza viruses, including matrix proteins of measles virus (MeV) and rabies virus (RABV). Additionally, we shortened TfR1 by substituting its extracellular domain and transmembrane sequence with an isoleucine zipper and the intracellular domain was truncated into various versions.

This part has been revised thoroughly and more details please refer to Major revision 6 described above.

**Reviewer #2:**

**Major Issues：**

*1.	Why use qRT-PCR to assess virus attachment instead of a plaque assay?*

Thank you for raising this critical point. In fact, based on our previous experience on quantitative measurement of virus titers, we find that qRT-PCR is superior to the plaque assay, given its high-throughput capability for a large number of samples in a very short time. While the plaque assay is visual but the process is tedious and time-consuming and the quantitative measurement is sometimes inconsistent. In this study, the assessment of viral attachment requires termination of the experiment at early infection stages to avoid interference from subsequent processes such as endocytosis and membrane fusion. qRT-PCR directly quantifies viral genomic RNA (vRNA) adsorbed to the cell surface, leveraging its high sensitivity and rapid quantification capabilities, which are optimal for precise detection at short time points. In contrast, plaque assays depend on the completion of the full viral replication cycle (typically ≥24 hours) and measure the infectivity of progeny virions, thereby failing to distinguish the biological effects of viral attachment from later invasion steps. Thus, this study employed qRT-PCR to specifically dissect the regulatory mechanisms governing the viral attachment phase. We have stated in the experimental methods why we used PCR instead of plaque assays in revised manuscript.

The updated manuscript is presented as follows: “The assessment of viral attachment requires termination of the experiment at early infection stages to avoid interference from subsequent processes such as endocytosis and membrane fusion. qRT-PCR directly quantifies viral genomic RNA (vRNA) adsorbed to the cell surface, leveraging its high sensitivity and rapid quantification capabilities, making it more optimal for precise detection at short time points compared to plaque assays.” (page 35)

*2.	Why were different multiplicity of infection (MOI) values used as opposed to a set standard MOI for all virus infection assays?*

Thank you for raising this critical point. In fact, for most virus infection assays in this study the same MOI (MOI = 0.5) was utilized except some extreme cases, such as the photo-crosslinking experiments which rely on high MOI (MOI = 50), the long-term (even 9 days) infection assay (MOI = 0.01) and the special sulfo-NHS-SS-Biotin-labeled IAVs for specific purpose (MOI=5). The photo-crosslinking experiment requires a higher viral load to increase receptor capture efficiency, whereas the longer infection period requires a lower viral dose to allow for extended cell survival. To avoid confusion caused by inconsistent MOI, we have provided explanations regarding the virus MOI in the revised manuscript.

The updated manuscript is presented as follows: “Given the robust affinity identified between TfR1 and matrix M1 in co-immunoprecipitation assay, a time-course analysis was employed to monitor the expression dynamics of M1 and TfR1 in infected A549 cells (MOI=0.01, a lower MOI was used to allow the cells to survive under prolonged viral infection conditions).” (page 8)

The updated manuscript is presented as follows: “A549 cells were seeded into 10 cm^2^ dishes at a density of 3×10^6^ cells 12 hours before photo-crosslinking. Then host cells were infected with DIBO-DAZ-Biotin labelled Influenza A virus (MOI = 50, to increase the capture rate of photo-crosslinking) and incubated at 4^o^C for 2 hours.” (page 34)

The updated manuscript is presented as follows: “To measure virus entry, cells were infected with IAV (MOI=5, increasing the virus MOI to detect viruses during transient infections) at 4^o^C for 1 hour and then transferred to 37°C for 1 hour.” (page 36)

*3.	While the data presented in figure 4 panels A-D are convincing, it could be stronger if the biological effects assayed were also evaluated at using qRT-PCR. There is the possibility that depending on how much plasmid is introduced that the effect shown in Western blot could be due to plasmid competition as opposed to an actual biological effect.*

Thank you for this valuable comment. Accordingly, we validated whether plasmid competition during the co-transfection of TfR1/SLC3A2/Vector plasmids with M1 plasmid under our experimental conditions would affect M1 mRNA transcription, which could influence the WB results. We used the mRNA transcription level of M1 when co-transfected with the Vector plasmid as the baseline. The results showed that although the mRNA transcription levels of M1 decreased when co-transfected with SLC3A2 or TfR1 plasmids, there was no significant difference between the M1+SLC3A2 and M1+TfR1 groups (**Figure S5G**). This indicates that under our experimental conditions, co-transfection of plasmids does not have a significant impact on the mRNA transcription levels across different experimental groups.

The updated manuscript is presented as follows: “Meanwhile co-transfection of the M1 plasmid with TfR1 or SLC3A2 plasmid had no influence on M1 transcriptional level (**Figure S5G**).” (page 9)

*4.	The introduction of the HDAC6 inhibitor tubacin offers another layer of intricate complexity to role of TfR1 in IAV particle packaging. Have the authors performed experiments seeing whether treating IAV-infected cells with tubacin yields similar results to IAV plasmids transfected into HEK293T cells?*

Thank you for this valuable comment. Accordingly, we have performed experiments - treating IAV-infected cells with tubacin and measured the viral titers by qRT-PCR. We found that compared to the control group, cells with added tubacin exhibited increased viral production, as shown in the revised **Figure S5D**. This suggests that tubacin may inhibit HDAC6, thereby preventing M1 protein degradation and promoting viral production.

The updated manuscript is presented as follows: “To further corroborate these findings, we performed parallel experiments in IAV-infected cells treated with Tubacin and observed comparable viral titer patterns via qRT-PCR analysis to those from IAV plasmid-transfected HEK293T cells (**Figure S5D**).” (page 9)

*5.	In figure 6 panel D, infecting cells with IAV instead of using IAV plasmids could provide more support that plasmid competition is not a factor impacting the results of TfR1’s effects on IAV replication.*

Thank you for this valuable comment. Accordingly, we have performed experiments - infecting cells with IAV instead of using IAV packing plasmids in **Figure S6B**. In this experiment, we selected the peptide (1-90-ZH, 1-67-ZH, 53-67-ZH) that contain the intracellular domain structure of TfR1, which we believe to be the key domain responsible for TfR1's degradation function. This approach prevents the use of full-length TfR1 (including the extracellular domain), which could facilitate viral entry into cells and lead to inaccurate experimental results. Experimental results show that overexpression of the TfR1 intracellular domain can effectively inhibit the CPE (cytopathic effect) induced by IAV infection.

The updated manuscript is presented as follows: “It was found that all of them predominantly existed as dimers and trimers (**Figure 6A**) and exhibited notable efficacy in degrading M1 protein in co-transfection and IAV infection **(Figure 6B** and **Figure S6B**), whereas the GST negative control showed no detectable impact on M1 degradation.” (page 10)

**Minor Issues：**

*1.	Which statistical analysis method was used to determine that the rescue of TfR1 and M1 in the presence of the proteasome inhibitor MG132 was significant as stated in the text? Statistical analysis to support that statement would strengthen the argument that the proteasome-aggresome pathway plays a role in TfR1-mediated degradation of M1.*

Thank you for this valuable comment. We have used mean gray value from Western blotting for statistical analysis, as shown in the revised **Figure 4D**.

*2.	There are inconsistencies in the text when discussing the different IAV strains and metabolically-labeled IAV particles. In the figure 2 legend for panels A and B, it is unclear whether wild-type IAV or metabolically-labelled IAV particles were used. In panel C of the same figure legend, the strains used were clearly stated.*

Thank you for raising this critical point. We have thoroughly revised this manuscript to make it consistent when wild-type IAV strains and metabolically-labeled IAV particles were referred. Unless otherwise specified, IAV refers to WSN, the wild-type A/WSN/1933 (H1N1) in the **Figure 2** legend.

The updated **Figure 2** legend is presented as follows: “**Figure 2.** Elucidation of host TfR1 as an IAV entry mediator critically for triggering viral endocytosis. (A) Western blot analysis examining the impact of TfR1 knockdown in A549 cells on IAV (A/WSN/1933 (H1N1))12 hours infection (MOI=0.5). Equal loading was confirmed by detecting GAPDH. (B) Immunofluorescent staining of A549 cells and cells with TfR1 knockdown 4h post -infectio (A/WSN/1933 (H1N1)) (MOI=0.5). (C) Assessment of the inhibitory effect of TfR1 knockdown on IAV infection (MOI=0.5) in A549 cells via RT-qPCR, spanning multiple subtype strains, including A/WSN/1933 (H1N1), A/PR/8/1934 (H1N1), and A/Aichi/2/1968 (H3N2). (D) Western blot characterization investigating the correlation between TfR1 expression levels in different cell lines and their susceptibility to IAV (A/WSN/1933 (H1N1)) infection (MOI=0.5). Equal loading was confirmed by detecting GAPDH. (E) Comparative evaluation of sialic acid versus TfR1 as potential receptors in mediating IAV entry. A549 cells transfected with TfR1/vector plasmid were subjected to neuraminidase (1:1000)/BSA treatment at 37°C for 2 hours before IAV (A/WSN/1933 (H1N1)) infection (MOI=0.5) (left). IAV were subjected to neuraminidase /BSA treatment 37°C for 2 hours before infecting A549 cells (right). Infected cells were lysed and quantified by RT-qPCR after 12 hours . (F) Comparative evaluation of TfR1 as a potential receptor in mediating IAV attachment versus endocytosis. A549 and TfR1-knockdown cells were incubated with IAVs (A/WSN/1933 (H1N1)) (MOI=10) or sulfo-NHS-SS-Biotin-labeled IAVs (A/WSN/1933 (H1N1)) (MOI=5) either at 4°C for 1 hour or then 37°C for 2 hours. Following washing, attached or internalized viruses were quantified by RT-qPCR or flow cytometry. (G) Flow cytometry analysis assessing TfR1 involvement in IAV entry, quantifying internalized biotin-SS-labeled IAVs (A/WSN/1933 (H1N1)) (MOI=5) in A549 cells, TfR1-knockout A549 cells, and CHO cells.”

*3.	For figure 2 panels F and G, the flow cytometry gating strategies used to curate and interpret data should be placed in the supplementary section or made available online.*

Thank you for this valuable comment. Accordingly, the flow cytometry gating strategies panels of **Figure 2** panels F and G have been placed in the Figure S2G. More details please refer to the revised manuscript.

*4.	Figure 2 panel E is confusing. It would be better to have two plots: one plot with the data on A549 cells incubated with neuraminidase and a second plot with the data on IAV incubated with neuraminidase. The data with Lec1 cells could be placed in the supplemental data section.*

Thank you for this valuable comment. Accordingly, **Figure 2** panel E have been separated into 3 plots: one plot with the data on A549 cells incubated with neuraminidase, a second plot with the data on IAV incubated with neuraminidase, as shown in the revised **Figure 2E**. The data with Lec1 cells were placed in the **Figure S2E**.

More details please refer to the revised manuscript.

*5.	The data in figure 3 panel A could be better curated. The smaller Western blot panels showing TfR1 expression with one IAV protein could be placed in the supplemental data section since the larger Western blot panel is descriptive enough.*

Thank you for this valuable comment. Based on another reviewer’s suggestion, we have repeated the co-immunoprecipitation experiments after 2 and 6 hours, respectively, post-infection at a MOI of 20. Accordingly, we have revised **Figure 3** panel A by replacement of the 6 h Western data in the supplemental data section while kept the 2 h data in the revised **Figure 3A**.

More details please refer to the revised manuscript.

*6.	The representative model in figure 6 panel F provides a helpful visual that ties in the key findings described in the paper.*

Thank you very much for the encouraging comment.

**Reviewer #3:**

**Major Issues：**

*1.	Results section 2.4: It is unclear what the role of HDAC6 is in the TfR1-mediated degradation of M1. During aggresome formation, proteasome activity is downregulated, which explains the inhibition of M1 degradation. Additionally, Trf1 and M1 co-localize with HDAC6 in the aggresome. However, based on our experience, when a protein is overexpressed, it typically aggregates in the aggresome after proteasome inhibition. The confusion lies in the application of Tubacin. Tubacin only interferes with HDAC6’s enzymatic activity, not its ubiquitin-binding ability, which regulates aggresomes. How can the authors conclude that the HDAC6-Ub pathway is affected? Furthermore, while Tubacin inhibits aggresome formation, this does not logically connect to the subsequent data (Fig. 4F to H). In Fig. 4G, the green aggregates are not aggresomes. Aggresomes should be unique within the cell. Furthermore, the figure legends are vague. What do the arrows indicate? What does the cyan color represent? These details should be clearly explained.*

Thank you for raising this critical point. We totally agree with the reviewer that it is the proteasome pathway rather than HDAC6-Ub pathway involved in the process of mutual TfR1 and M1 degradation. Given the prior disclosure of the involvement of the host proteasome and aggresome systems in M1 degradation [31] and current identification of the direct interactions between TfR1 and NA as well as their reciprocal degradation, we are wondering whether these pathways influence the regulation of TfR1 levels either. We have designed experiments to test TfR1-mediated M1 degradation in the presence of a proteasome inhibitor (MG132), an HDAC6 inhibitor (Tubacin, inhibition of HDAC6 by tubacin could regulate the aggresomes, doi:10.1073/pnas.0503221102) or a lysosome inhibitor (HCQ). We found MG132 exerted significant inhibition followed by Tubacin to a lesser extent but not HCQ. This suggests that the proteasome pathway and HDAC6 but not lysosome are engaged in this degradation process. Additionally, to further identify aggresomes, we have labeled M1 and TfR1 and updated the **Figure 4G**. As discussed in the study limitation section, multiplexed mechanisms and factors rather than just proteasome- and HDAC6-mediated pathway may engage in the process. Regarding the reciprocal degradation of TfR1 and M1, further molecular insights into the reciprocal degradation of TfR1 and M1 remains to be elucidated and is necessary. Therefore, we have toned down mechanistic description in the revised manuscript. Additionally, the figures and figure legends have been re-written to clarify our points based on the reviewer’s comments.

The updated manuscript is presented as follows: “Building upon prior research indicating the involvement of the host proteasome and aggresome systems in M1 degradation [31], we tested whether these pathways influence the regulation of TfR1 levels. Transfection of the IAV plasmid complex and TfR1 into 293T cells in the presence of MG132, a proteasome inhibitor, indicated a significant rescue in the expression levels of both TfR1 and M1, while HA and NA proteins remained unaffected (**Figure 4D**). Additionally, Tubacin, an HDAC6 inhibitor, but not the lysosome inhibitor HCQ, also elicited a notable increase in TfR1 and M1 levels, although to a lesser extent than the effect seen with MG132 (**Figure 4E**, **Figure S5C** and **Figure S5E**). To further corroborate these findings, we performed parallel experiments in IAV-infected cells treated with Tubacin and observed comparable viral titer patterns via qRT-PCR analysis to those from IAV plasmid-transfected HEK293T cells (**Figure S5D**). The significant inhibition of MG132 followed by Tubacin but not HCQ suggests engagement of the proteasome pathway and HDAC6 but not lysosome in this degradation process. Subsequent laser confocal microscopy experiments unveiled that co-transfected TfR1 and M1 formed protein aggregates within host cells (**Figure 4F-G**), co-localizing with ubiquitin and HDAC6; this was supported by the in vitro aggregation study (**Figure 4H**). From these observations, we infer that the mutual degradation of interacting M1 and TfR1 proteins involves an interplay between HDAC6-mediated protein degradation and ubiquitin-proteasome pathways, and the formation of the M1-TfR1 as a misfolded complex triggers their concurrent degradation.” (page 7)

*2.	In Fig. 2A, the knockdown of TfR1 inhibits IAV infection, indicating that TfR1 is essential for viral infection, which seems to suggest a proviral role. However, at the end of the results section, the authors claim that TfR1 acts as an antiviral agent by degrading the M1 protein. What is the exact role of TfR1? Is the extracellular domain necessary for virus infection while the intracellular domain is responsible for antiviral M1 degradation? Additionally, Fig. 1D shows that high levels of TfR1 correspond to high levels of M1 and increased IAV susceptibility. Does this contradict the later finding that high TfR1 levels lead to M1 degradation?*

Thank you for raising those critical points and we are sorry for the vague description that makes our points confusing. As the reviewer commented, our main point is that TfR1 plays dual functions in virus infection, initially serving as an endocytosis trigger by engaging its extracellular domain with the viral envelope NA, and subsequently functions as a matrix degradator by interacting via its intracellular sequence with the viral matrix M1. Given the IAV matrix protein as an important component for progeny virus production, TfR1-mediated degradation of M1 may provide an exciting avenue for the development of novel antivirals. This is supported by the broad-spectrum effect of TfR1-mediated degradation on the viral matrix protein and on IAV progeny virus production from TfR1-transfected cells and infected cells with IAV in a dose-dependent manner (**Figure 6D**). We have re-written this part to clarify our points in the revised manuscript.

The updated manuscript is presented as follows: “Given the IAV matrix protein as an important component for progeny virus production, TfR1-mediated degradation of M1 provides an exciting avenue for the development of novel antivirals. We first evaluated the broad-spectrum effect of TfR1-mediated degradation on the viral matrix protein, spanning antigenically distinct influenza strains. We found significant degradation of matrix proteins by TfR1 extended to A/Puerto Rico/8/1934 (H1N1), A/Aichi/2/1968 (H3N2), and A/Anhui/1/2013 (H7N9), attributed to 92% sequence homology in M1 among these distantly related influenza strains (**Figure 6C**). The influence of TfR1-mediated M1 degradation also displayed a clear dose-dependent inhibition of IAV progeny virus production from IAV plasmids complex-transfected cells and infected cells with IAV (**Figure 6D**). Furthermore, investigations revealed that TfR1-mediated degradation extended beyond influenza viruses, as co-transfection with TfR1 led to degradation of matrix proteins of measles virus (MeV) and rabies virus (RABV) in a dose-dependent manner (**Figure 6C** and **Figure 6E**). Hence, the discovery of viral matrix protein degradation by TfR1 holds promise for the development of degradative antiviral agents that transcend the scope of influenza viruses.” (page 10)

Additionally, we provide a representative model in **Figure 6F** that provides a helpful visual that ties in the key findings - one stone two birds function of TfR1 exploited by influenza virus for IAV entry - described in the paper.

*3.	That the authors use a 24 hpi time point to co-IP the TFr1 and M1 is confusing. 24 hpi is a very late time point of infection and has little to do with IAV entry, but more with assembly and release.*

Based on editor’s and reviewer’s comments, we have repeated the co-immunoprecipitation experiments after 2 and 6 hours, respectively, post-infection at a MOI of 20. We found that NA and M1 were still co-immunoprecipitated by TfR1 to a little different extent; this suggests that within 24 hours the interaction of TfR1 with NA and M1 still exists. We revised the manuscript accordingly，more details please refer to the revised manuscript.

The updated manuscript is presented as follows: “Subsequently, co-immunoprecipitation experiments were conducted using lysates from infected A549 cells to identify viral components interacting with TfR1. We found viral NA and M1 proteins were selectively co-immunoprecipitated with the anti-TfR1 antibody at either 2, 6 or even 24 hours post-infection, whereas HA and M2 proteins didn’t exhibit such interaction (**Figure 3A** and **Figure S3F**). These interactions between TfR1 and NA, as well as M1, were further confirmed through co-immunoprecipitation using anti-NA and anti-M1 antibodies. In contrast, neither anti-HA nor anti-M2 antibodies co-immunoprecipitated with TfR1.” (page 6)

*4.	Is the extracellular domain necessary for virus infection while the intracellular domain is responsible for antiviral M1 degradation.*

We are sorry for the vague description that makes our points confusing. As discussed above, TfR1 plays dual functions in virus infection, initially serving as an endocytosis trigger by engaging its extracellular domain with the viral envelope NA, and subsequently functions as a matrix degradator or viral uncoating by interacting via its intracellular sequence with the viral matrix M1. Given the IAV matrix protein as an important component for progeny virus production, TfR1-mediated degradation of M1 provides an exciting avenue for the development of novel antivirals. More details please refer to above reference.

Additionally, we have clarified our points through this manuscript title “TfR1 facilitates influenza virus endocytosis and uncoating by interacting with NA and M1 via extracellular and intracellular domains.”

*5.	Virus internalization assays (Page 34) were done in cell suspension with DMEM + 1% FCS. FCS is blocks IAV binding and infectivity massively, and at extreme low concentrations blocks CME, re-routes the virus to a dynamin-independent macropinocytic pathway (de Vries et al., 2023, was not cited). The experiments containing NH4Cl were not appeared to be buffered at pH 7.4 with HEPES (it is not clarified in the methods). How does 0.05% Trypsin affect surface TfR1 levels? See De Vries et al., (2011) Plos Path https://doi.org/10.1371/journal.ppat.1001329 Dissection of the Influenza A Virus Endocytic Routes Reveals Macropinocytosis as an Alternative Entry Pathway. The conclusion in Fig. 2F stating, “These findings suggest that TfR1 is more crucial for IAV endocytosis than for initial attachment to the cell surface. Consistently, further investigations into viral internalization were conducted using A549 cells,”is overinterpreted. Attachment and endocytosis are not independent steps; in fact, endocytosis follows attachment. Therefore, the observation of a greater inhibitory effect on endocytosis may be due to inefficient attachment, with the difference being amplified by the biological cascade.*

Thank you for raising this critical point and we totally agree with the reviewer. In fact, the entry of Influenza A viruses into host cells relies on a multifaceted, highly choreographed, multi-step process. However, this continuous process has been artificially defined into several stages: initial viral attachment to host cells, penetration of the host cell membrane, fusion between viral and endosomal membranes, uncoating of the viral matrix to release viral RNAs into the cytoplasm and so on. Given multiple cellular factors and host sialic acids involved in initial viral entry and sialic acid is not a requisite for effective IAV infection in GnT1-deficient CHO 15B and HEK 293S cells [doi: 10.1073/pnas.1200987109.], it suggests that while sialic acid may facilitate initial viral attachment to the cell membrane, it does not always guarantee efficient entry. The same situation occurred in this study as a ~25% reduction in virus attachment on A549 cells upon TfR1 knockdown, which led to 70-80% reduction in WSN infection.

This suggests that TfR1 has less effect on virus attachment but significant effect on entry; therefore, we designed experiments to scrutinize TfR1's involvement in virus entry, either attachment or endocytosis, despite of as an independent step. We have carefully designed the experiments, such as incubation of A549 cells with IAVs at 4°C for one hour to make efficient attachment, adding 1% FCS in the assay medium to exclude unspecific binding and adding endocytosis inhibitor NaN_3_ as a control as well as three time washing post incubation.

We have toned down this conclusion “These findings suggest that TfR1 is more crucial for IAV endocytosis than for initial attachment to the cell surface” as “Thus, it seems likely that TfR1 is more crucial for IAV endocytosis than for initial attachment to the cell surface, despite they are not independent steps.” Additionally, we have clarified the pH buffering in NH_4_Cl experiments and 0.05% Trypsin effect on surface TfR1 levels by flow cytometry analysis in the revised manuscript in **Figure S2B**. (page 5)

We would like to reiterate that we highly appreciate the reviewer's comment that the virus may utilize different infection pathways under different conditions. We also appreciate the research papers provided by the reviewer. Therefore, in the discussion section, we have highlighted the research limitations caused by the potential use of different infection pathways by the virus under varying experimental conditions, and we have cited important references to support this. (doi: 10.1371/journal.ppat.1001329., doi: 10.1073/pnas.2304992120., doi: 10.1128/jvi.00602-23.) in our discussion.

The updated discussion is presented as follows: “Furthermore, in addition to receptor-dependent viral entry, viruses may utilize alternative infection pathways, such as the dynamin-independent macropinocytic pathway [79-81], under different conditions. This highlights the complexity of viral infection mechanisms and underscores the need for further investigation.”

The updated manuscript is presented as follows: “The subsequent focus of our investigation was to scrutinize TfR1's involvement in virus entry, either attachment or endocytosis. To assess the role of TfR1 in viral attachment, A549 cells and TfR1-knockdown A549 cells were incubated with IAVs (MOI=10, with culture medium containing 1% FBS to exclude unspecific binding) at 4°C for one hour. After washing three times, we quantified the attached viruses using RT-qPCR and found a ~25% reduction in virus attachment in TfR1-knockdown A549 cells compared to those treated with negative siRNA, highlighting the significant role of TfR1 in the initial attachment of IAV particles to host cells. To evaluate TfR1's role in the endocytosis of attached virus particles, we transferred the aforementioned cells from 4°C to 37°C and incubated them for an additional two hours with or without NaN_3_, a viral endocytosis inhibitor. RT-qPCR analysis revealed that TfR1-knockdown cells exhibited a ~70% reduction in the number of internalized viruses compared to control cells (**Figure 2F** and **Figure S2G**). The viral internalization was further investigated by exposure of A549 cells, TfR1-knockout A549 cells, and CHO cells to sulfo-NHS-SS-Biotin-labeled IAVs [37] (MOI=5) under identical conditions. Flow cytometry analysis, using AF488-conjugated streptavidin [36], showed an ~80% reduction in mean fluorescence intensity in TfR1-knockout cells compared to parental A549 cells (**Figure S2B**). This reduction was comparable to that observed in CHO cells, which inherently lack TfR1 expression. Moreover, overexpression of TfR1 in TfR1-knockout A549 cells restored IAV endocytosis (**Figure 2G**, **Figure S2B** and **Figure S2G**). Thus, it seems likely that TfR1 is more crucial for IAV endocytosis than for initial attachment to the cell surface, despite they are not independent steps.” (page 5)

**Minor Issues：**

*1.	In Fig. 4B, the GAPDH levels are not consistent.*

Thank you for raising this critical point and we have repeated this experiment and obtained consistent GAPDH levels in the Western blot.

*2.	References; the main text cites Schmolke’s work (Mazel-Sanchez B et al., 2023) as [25], however, in the References it is [24].*

Thank you for raising this critical point and we have corrected this mistake in the revised manuscript.

*3.	Overinterpretation of the data. End of the Introduction “This mechanism unveils the long-…” should be toned down.*

Thank you for raising this critical point and we have re-written this sentence as “This mechanism unveils the potential factor responsible for triggering matrix protein 1 (M1) degradation [29], potentially involving proteasome and HDAC6 mediated pathways [30-32]—a pivotal step following HA/endosomal fusion to release viral RNAs into the cytoplasm.” (page 3)

*4.	For Fig. S1D and S2D, a quantitative analysis of co-localization should be performed.*

Thank you for this valuable comment. Accordingly, quantitative analysis of co-localization in **Figure S1D** and **S2D** has been conducted as shown in the revised manuscript.

*5.	Figure S2D is unfortunately not a convincing display of co-localization. For one thing, there is excessive NA signal (saturated) due to virus overload. Claims of co-localization require higher resolution to enable single virus resolution and performed at an optimal virus particle number per cell.*

Thank you for raising this critical point. Accordingly, we have adjusted the background (due to black-and-white imbalance) in the images and a higher resolution to depict the distribution of virus particles per cell has been added in the revised manuscript.

*6.	Fig. 2E is not adequately explained. Is TfR1 overexpressed? What is a Lec1 cell? Please clarify in the figure legend. What is the concentration of Neuramindase used.*

Thank you for this valuable comment. Lec1 cells are CHO cells deficient in N-acetylglucosaminyltransferase I (GnT-I), an enzyme critical for normal N-glycosylation. Specifically, the absence of GnT-I in Lec1 cell prevents the addition of N-glycan chains to proteins, resulting in a characteristic lack of glycosylation. Accordingly, Figure 2E have been separated into 3 plots: one plot with the data on A549 cells incubated with neuraminidase, a second plot with the data on IAV incubated with neuraminidase (1:1000), as shown in the revised Figure 2E. The data with Lec1 cells were placed in **Figure S2E**. More details please refer to the revised manuscript. (page 4)

*7.	In Fig. 3F, the interface analysis, particularly regarding hydrogen bonds based on docking simulations, is not recommended. Such information should be derived from the actual 3D structure, as predicted models cannot accurately indicate the positions of side chains. Additionally, the parameters and evaluations of the docking simulation should be provided.*

Thank you for this valuable comment and this is indeed one limitation in this study. Given the direct interaction between TfR1 and NA proteins identified by a variety of methods, the precise region of this interaction and the exact binding configuration has yet to be determined and warrants further rigorous investigation. Based on reviewer’s comments, the hydrogen bond analysis of **Figure 3F** has been removed, and only one schematic diagram has been retained. Additionally, the parameters and evaluations of the docking simulation have been provided in the revised manuscript.

The updated manuscript is presented as follows: “**Docking analysis**: Protein structures of influenza virus neuraminidase (NA; PDB ID: 6Q23) and transferrin receptor 1 (TfR1; PDB ID: 1SUV) were retrieved from the Protein Data Bank (https://www.rcsb.org/). Molecular docking simulations were performed using the HDOCK server (http://hdock.phys.hust.edu.cn/), a web-based platform for protein-protein interaction prediction. The docking protocol utilized default parameters to generate an ensemble of putative binding conformations. The most suitable conformation, was selected for further structural analysis. Protein-protein interaction was visualized using PyMOL to identify binding conformation involved in the NA-TfR1 interaction.” (page 36)

*8.	In Fig. 4A, how did the authors maintain A549 cells in culture for 9 days?*

Thank you for raising this critical point. Given the observations of TfR1-mediated M1 degradation in the preliminary data, we would like to verify this phenomenon by careful design of an experiment to monitor WSN-infected A549 cells as long as possible. Thus, A549 cells at a low density (50%) were seeded and infected with WSN viruses at a low MOI (MOI=0.01); the culture medium was refreshed every day to maintain A549 cell viability.
