# Supplementary material for: TfR1 facilitates influenza virus endocytosis and uncoating by interacting with NA and M1 via extracellular and intracellular domains
Source: PLoS Pathog. 2025 Oct 10;21(10):e1013511. doi: 10.1371/journal.ppat.1013511 (PMC12533913; doi:10.1371/journal.ppat.1013511)
Supplement: Attachment — Submitted filename: Response_to_Reviewers_auresp_2.docx [file ppat.1013511.s011.docx]

Dear Editors of PLOS Pathogens,

We sincerely appreciate the time and effort you and the reviewers have dedicated to evaluating our manuscript, "TfR1 facilitates influenza virus endocytosis and uncoating by interacting with NA and M1 via extracellular and intracellular domains" (PPATHOGENS-D-24-01941). We are grateful for the constructive feedback, which has significantly strengthened our work. Below, we provide point-by-point responses to the reviewers’ comments, with revisions highlighted in the manuscript. We hope these changes address all concerns and further enhance the manuscript’s clarity and impact.

***Editor’s comments:***

*They request for inclusion of more controls to classify the structures as HDAC6/Ub/aggresomes. For example, including gamma tubulin could help to clarify whether these structures are found in close vicinity of MTOC.*

Thank you for your valuable suggestion. We agree that additional controls would strengthen our characterization. As suggested, we performed co-localization experiments with γ-tubulin, a marker for the microtubule-organizing center (MTOC). The results (now in **Figure 4G**) confirm that TfR1-M1 aggregates localize to the MTOC region, supporting their identification as aggresomes. We appreciate this insightful suggestion.

*Also the observed punctate staining pattern of M1 appears independent of TfR expression and typically, M1 expression would be expected to be diffuse. Could you please clarify?*

Thank you for your valuable comment. We acknowledge that M1 can exhibit diffuse or multimeric patterns. In our original manuscript, the M1 signal was indeed diffusely distributed, but due to suboptimal black-and-white balance, it may have appeared more like multimeric patterns. To minimize confusion, we now highlight diffuse M1 signals in the revised **Figure 4G**.

**Reviewer #3:**

**Major Issues：**

*1.	HDAC6 and Tubacin experiments. Unfortunately, there are several shortcomings with the interpretation of HDAC6 and the aggresome pathway in TfR1-mediated M1 degradation. It is unclear why tubacin would prevent degradation of M1. Overexpression of the proteins under proteasome inhibition conditions commonly leads to protein aggregation. Therefore, the co-localization of ubiquitin with TfR1 and M1 shown in Fig. 4F provides weak evidence for the hypothesis proposed on p.27 that TfR1 induces misfolding of M1, subsequently directing it toward the ubiquitin-proteasome pathway. Could it be that ubiquitinated TfR1 directly targets M1 for degradation?*

Thank you for raising this critical point. We referenced a PNAS study (doi:10.1073/pnas.0503221102) showing that tubacin disrupts HDAC6-dynein binding, preventing aggresome formation. While our data align with this mechanism, we acknowledge that further validation is needed. Importantly, our observed TfR1-M1 aggregates formed without proteasome inhibition. To address spatial specificity, we included γ-tubulin co-localization data (**Figure 4G**), confirming MTOC association.

*2.	Proviral and antiviral roles of TfR1*

*The authors’ response indicates that TfR1-mediated degradation of M1 reduces progeny virus production, citing dose-dependent inhibition shown in Fig. 6D. However, co-transfection of multiple plasmids confounds the experiment - increasing the amount of one plasmid (TfR1) could affect transfection or expression efficiency of the other plasmids.*

*To support the authors’ conclusion, the following experiments are suggested:*

*• Include a suitable control experiment transfecting equal amounts of a neutral plasmid (e.g. SLC3A2 plasmid) to rule out non-specific competitive effects.*

*• Perform virus production assays using TfR1-knockdown or KO cells. Additionally, the experiment shown in Fig. S2F measures viral genome replication during a single-cycle infection, which does not provide direct evidence of progeny virus production inhibition by M1 degradation. Genome replication and progeny virus production/release are distinct steps; thus, an experiment specifically assessing progeny virus titers is necessary to support author’s claims.*

Thank you for raising this critical point. We apologize for the unclear methodology. The total plasmid DNA (1.5 µg) was kept constant, with TfR1 amounts balanced by a neutral control (SLC3A2). The revised text clarifies as A549 cells were co-transfected with 1.5 µg total DNA (12 IAV plasmids + variable TfR1/SLC3A2 ratios). Virus production was quantified at 48 h post-transfection by RT-qPCR.

The updated manuscript is presented as follows:

"TfR1-mediated M1 degradation suppresses IAV progeny production in a dose-dependent manner. A549 cells were co-transfected with a total of 1.5 μg plasmid DNA, consisting of 12 IAV packaging plasmids and increasing amounts of TfR1 plasmid, balanced with the control plasmid SLC3A2 to maintain consistent total DNA across conditions. Viral titers were measured by RT-qPCR at 48 h post-transfection and normalized to untransfected controls."

*3. The role of TfR1 in IAV endocytosis*

*In Figure 2F, the flow cytometry data are convincing. However, for RT-qPCR-based quantifications it is unclear to the reviewer how they removed extracellular virus particles before measuring the genome of internalized viruses. If extracellular viruses were not removed, how did they distinguish between extra- and intra-cellular viruses?*

Thank you for raising these critical points. In **Figure 2F**, the attachment data (left panel) were measured by RT-qPCR, while the endocytosis data (right panel) were assessed using flow cytometry. For the attachment assay, we allowed IAV to bind to the cell surface at 4 °C for 1 hour, a condition under which the virus remains on the plasma membrane and does not undergo internalization. This setup allows RT-qPCR to reflect primarily surface-bound viral genomes, without contribution from internalized virus. We have clarified this point in the revised manuscript.

The updated legend is presented as follows: “Comparative evaluation of TfR1 as a potential receptor mediating IAV attachment and endocytosis. A549 and TfR1-knockdown cells were incubated with either unlabeled IAVs (A/WSN/1933 (H1N1), MOI = 10) or sulfo-NHS-SS-Biotin-labeled IAVs (MOI = 5) at 4 °C for 1 hour (to allow attachment) or subsequently shifted to 37 °C for 2 hours (to allow endocytosis). After washing, attached viruses were quantified by RT-qPCR, and internalized viruses were measured by flow cytometry.”

**Minor Issues**

*1.	Figures S1D and S2D (co-localization analysis)*

*Clearly describe the method used for quantifying colocalization (Pearson’s correlation coefficient). The observed high correlation is most likely due to using a low magnification for the confocal microscopy.*

*They should add an additional control using an unrelated membrane protein such as SLC3A2 to validate the significance of the observed colocalization.*

Thank you for raising these critical points. We appreciate these insightful comments regarding our colocalization analysis. we have now clarified this point in the revised manuscript as following:

**Methodological Clarification:**

1. Pearson's correlation coefficients in **Figures S1D** and **S2D** were calculated using ImageJ
2. For **Figure S2D s**pecifically, analysis was performed on zoomed-in regions to accurately assess local colocalization
3. We have updated the figure legend to explicitly describe this methodology

**Additional Control Experiment:**

1. As recommended, we included SLC3A2 as an unrelated membrane protein control
2. This validation has been added as **Figure S1E** in the revised manuscript

**Updated Figure Legend:**

Colocalization analysis of NA with TfR1 and clathrin-mediated pathway components (TTP/CLTA) during IAV endocytosis. Color scheme: NA (blue), TfR1 (green), TTP/CLTA (red), nuclei (cyan). Scale bars: 50 μm. Pearson's coefficients (calculated from zoomed-in regions): NA-TfR1 = 0.672, NA-TTP = 0.689, NA-CLTA = 0.701, TfR1-CLTA = 0.744.

*2.	Neuraminidase concentration (Fig. 2E): Clarify the neuraminidase concentration used in units or μg/mL, not in dilution factor, for objectivity. Detailed methods information is lacking.*

Thank you for your thorough review and valuable feedback. We apologize for any lack of clarity in the original description. The neuraminidase concentration used in the experiments was 50 units/mL. We have revised the manuscript to present this information more clearly and objectively.

The updated section now reads as follows: “**Comparative evaluation of sialic acid versus TfR1 as potential receptors in mediating IAV entry.** A549 cells transfected with TfR1/vector plasmid were treated with neuraminidase (50 units/mL) or BSA at 37°C for 2 hours before infection with IAV (A/WSN/1933 (H1N1), MOI = 0.5) (left panel). Alternatively, IAV was pretreated with neuraminidase or BSA at 37°C for 2 hours before infecting A549 cells (right panel). After 12 hours, infected cells were lysed and quantified by RT-qPCR.”

*3.	Fig.4F & 4G: M1 should be diffused distrubted throughout cell upon single expression. It is unclear whether the cells were MG132-treated and there is no ZsGreen-only control. Right-most column: appears to be an aggregate, rather than a MTOC-associated aggresome.*

Thank you for raising these important points. The cells in **Figures 4F** and **4G** were not treated with MG132. We appreciate your suggestion to differentiate between general aggregates and MTOC-associated aggresomes.

To further investigate this, we conducted additional co-localization experiments with an MTOC marker (γ-tubulin). The results, now included in the revised manuscript as **Figure 4G**, confirm that the TfR1-M1 aggresome localizes to the MTOC region.

*4.	Figure 1E: Does this figure indicate binding of TfR1 to viral components even without UV crosslinking? Please clarify the intended conclusion from Fig. 1E.*

We sincerely appreciate your thorough review of our manuscript and apologize for any lack of clarity in our original explanation. In **Figure 1E**, we aimed to demonstrate that the UV-crosslinked group (right panel) exhibited a distinct band shift in TfR1 compared to the non-UV group (left panel). This shift confirms successful capture of TfR1 by viral components carrying photo-reactive crosslinkers on the IAV surface. We have clarified this point in the revised figure legend, which now reads:

**“Analysis of photo-crosslinked products from A549 cells infected with IAV (MOI=50) pre-labeled with DIBO-DAZ-Biotin or left untreated.** Cells were incubated with virus for 1 h on ice, then shifted to 37°C for 5 min. Samples were either exposed to UV irradiation (+) or not (−) for 20 min. Crosslinked proteins were enriched using streptavidin agarose, separated by 8% SDS-PAGE, and detected by western blotting with anti-HA, anti-NA, or anti-TfR1 antibodies. The observed mobility shift of TfR1 in the UV-treated group indicates successful photo-crosslinking between TfR1 and viral components.”

*5.	Figures 2A, 2D, S2C, and others: Virus entry/infection was measured by the expression of intracellular M1 protein, yet the authors claim that M1 is degraded upon cell entry via TfR1. This method is problematic and confusing because differences in M1 levels might reflect differential degradation rather than differences in virus entry. We recommend quantifying virus entry/infection using nucleoprotein (NP), a viral protein unaffected by TfR1 expression according to the authors’ own data.*

We sincerely appreciate your careful review of the figures and your insightful comments. We understand your concern regarding the potential confounding effect of M1 degradation on virus entry measurements and appreciate the opportunity to clarify this point.

In our study, viral entry/infection was assessed at early time points (6–12 hours post-infection), while M1 degradation was typically observed at later stages (48–72 hours post-infection). Importantly, if TfR1-mediated degradation were significantly affecting M1 levels during the entry stage, we would expect TfR1 knockdown to increase M1 expression. However, we observed the opposite effect - a decrease in M1 signal upon TfR1 knockdown at early time points. This finding strongly suggests that TfR1 primarily facilitates viral entry or early infection rather than mediating M1 degradation.

We completely agree that using HA, a viral protein unaffected by TfR1 levels, would provide a more specific readout. To address this concern and improve clarity, we have now included anti-HA western blot data in **Figure 2A** as an additional control.

*6.	Figure 2C: Clarify the exact hours post-infection when these cells were collected.*

We appreciate this helpful suggestion. In response, we have now specified the post-infection time point in the figure legend.

The updated legend reads: "Evaluation of TfR1 knockdown's inhibitory effect on IAV infection (MOI = 0.5) in A549 cells. Viral RNA levels were quantified by RT-qPCR at 12 hours post-infection across multiple strains: A/WSN/1933 (H1N1), A/PR/8/1934 (H1N1), and A/Aichi/2/1968 (H3N2)."

*7.	Figure S5D: Please indicate which data correspond to the "IAV plasmid transfected HEK293T cells" mentioned in the main text.*

We sincerely appreciate this insightful comment and apologize for our oversight in not referencing **Figure 4E**. We have now included this important reference in the revised manuscript text:

"To further validate these findings, parallel experiments in IAV-infected cells treated with Tubacin revealed viral titer patterns by qRT-PCR that were consistent with those observed in IAV plasmid-transfected HEK293T cells (**Figure 4E** and **Figure S5D**)."

*8.	Page 10 text and Figure S6C mismatch: The statement, “Further truncations demonstrated that expression of the intracellular and transmembrane domain...” does not match the data shown in Fig. S6C. Please correct or clarify this discrepancy.*

We sincerely appreciate this important observation and apologize for the error in our original figure reference. Upon careful review, we confirm that **Figure S6A** (not **Figure S6C**) supports this finding. The corrected text now reads:

"Additional truncation analysis revealed that the intracellular and transmembrane domains (Intra+Tm (1-88)) retained M1 degradation activity (**Figure 5C** and **Figure S6A**)."

*9.	Figure 5C and 5D (TfR1 truncated mutants): The subcellular localization of truncated TfR1 mutants (e.g., Δ1-63, ΔTM) needs to be shown. At least for mutants affecting M1 degradation capability, localization data are essential to confirm whether functional differences might be due to altered cellular localization. The M1 blot is missing.*

We sincerely appreciate these insightful observations regarding the importance of subcellular localization in evaluating TfR1 truncation mutants. In response, we performed confocal microscopy analysis of the ΔTM and Δ1-67 mutants, with these new data now included as **Figure S6E** in the revised manuscript.

Our results demonstrate that these truncated mutants maintain localization patterns comparable to wild-type TfR1. This finding suggests that the observed variations in M1 degradation activity are not attributable to differences in subcellular distribution.

*10.	Figure 6B (label clarification): Please clarify what the term "cyto" means in Fig. 6B. Is this equivalent to the "intracellular domain" described in Fig. 5D?*

We appreciate this helpful observation. To maintain consistency with our terminology, we have revised **Figure 6B** by replacing "cyto" with "intra" to clearly indicate the intracellular domain, matching the description used in **Figure 5D**. This change ensures uniform terminology throughout the manuscript.
